# Supplementary material for: HOXA13 in etiology and oncogenic potential of Barrett’s esophagus
Source: Nat Commun. 2021 Jun 7;12:3354. doi: 10.1038/s41467-021-23641-8 (PMC8184780; doi:10.1038/s41467-021-23641-8)
Supplement: Supplementary file 1 — Supplementary Information [file 41467_2021_23641_MOESM1_ESM.pdf]

HOXA13 in etiology and oncogenic potential of Barrett's esophagus

Vincent T. Janmaat, Kateryna Nesteruk, Manon C.W. Spaander, Auke P. Verhaar, Bingting Yu, Rodrigo A. Silva, Wayne A. Phillips, Marcin Magierowski, Anouk van de Winkel, H. Scott Stadler, Tatiana Sandoval-Guzmán, Luc J.W. van der Laan, Ernst J. Kuipers, Ron Smits, Marco J. Bruno, Gwenny M. Fuhler[#], Nicholas J. Clemons, & Maikel P. Peppelenbosch[#].

Supplementary figures

a

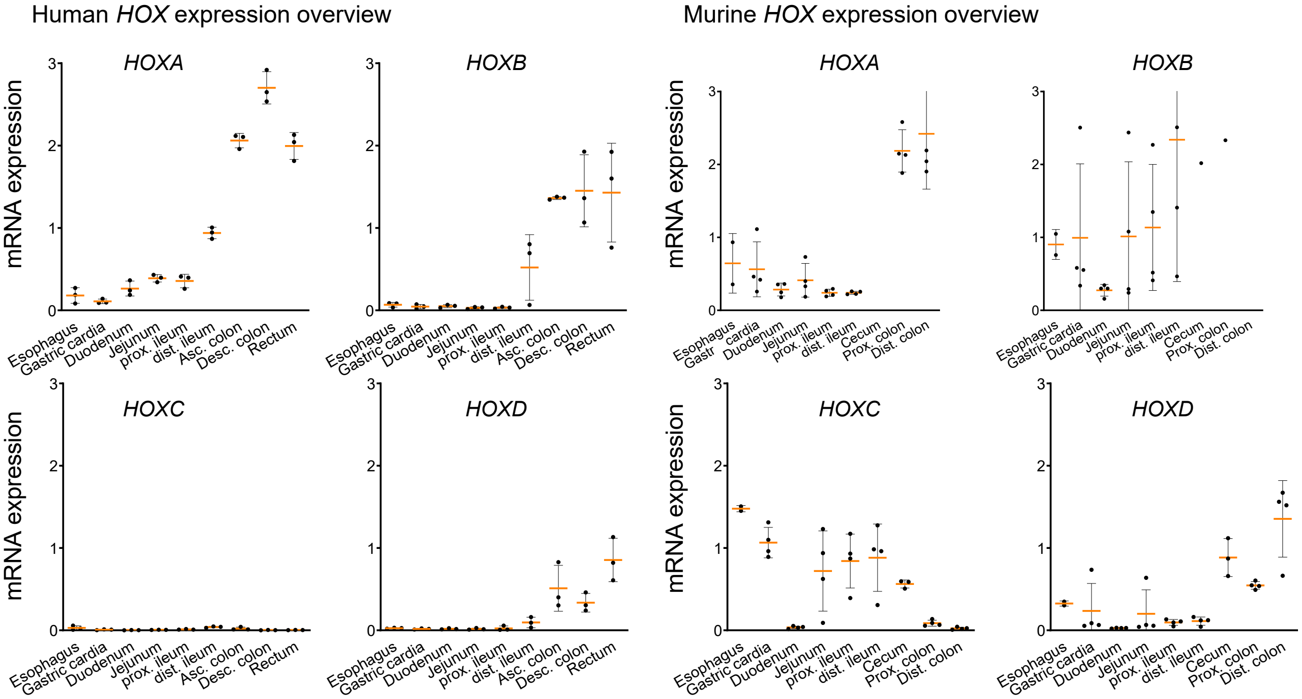

b

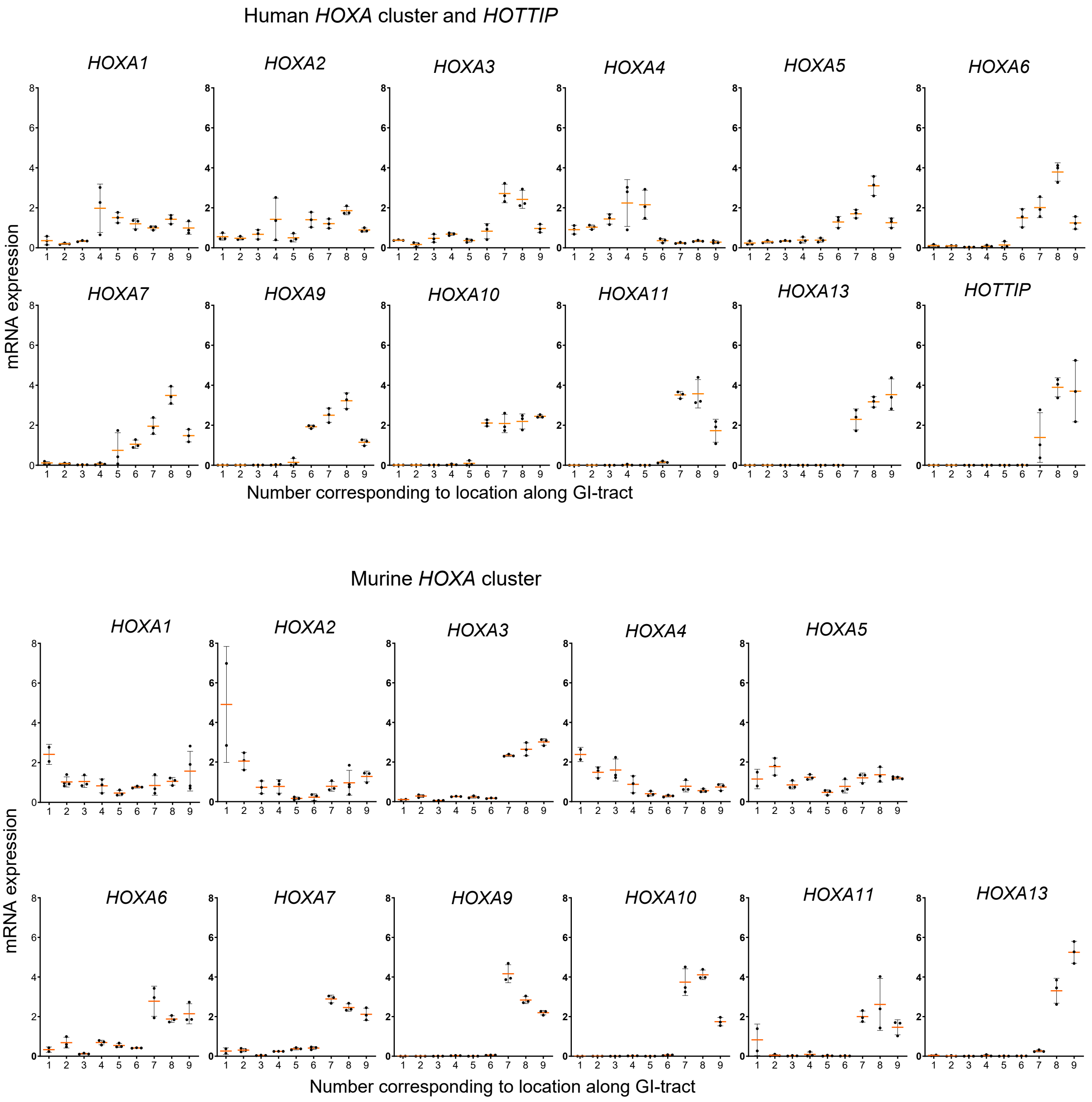
Human *HOXA* cluster and *HOTTIP*
HOXA1
HOXA2
HOXA3
HOXA4
HOXA5
HOXA6
HOXA7
HOXA9
HOXA10
HOXA11
HOXA13
HOTTIP
mRNA expression
Number corresponding to location along GI-tract
Murine *HOXA* cluster
HOXA1
HOXA2
HOXA3
HOXA4
HOXA5
HOXA6
HOXA7
HOXA9
HOXA10
HOXA11
HOXA13
mRNA expression
Number corresponding to location along GI-tract

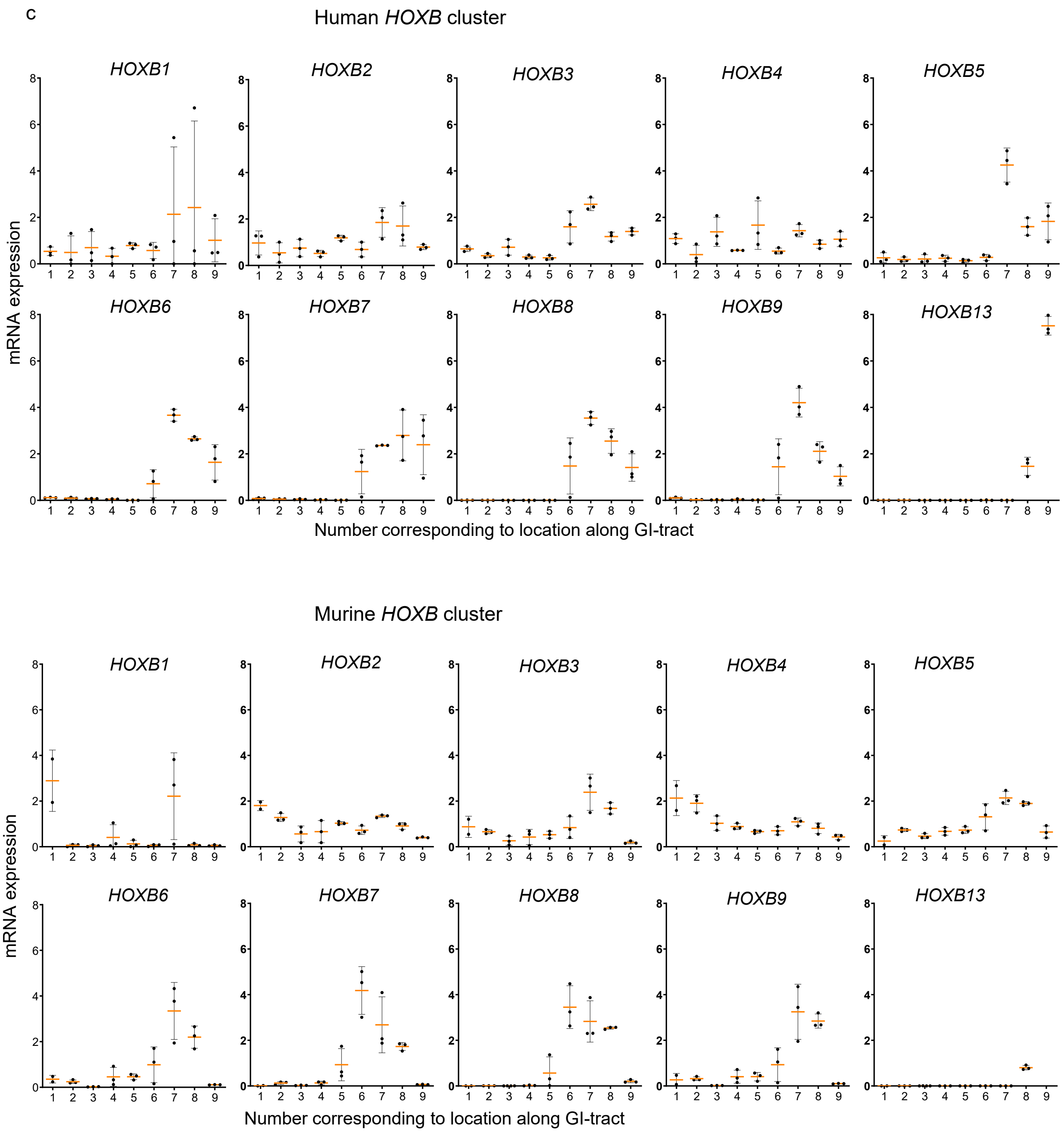
c
Human HOXB cluster
HOXB1
HOXB2
HOXB3
HOXB4
HOXB5
HOXB6
HOXB7
HOXB8
HOXB9
HOXB13
mRNA expression
Number corresponding to location along GI-tract
Murine HOXB cluster
HOXB1
HOXB2
HOXB3
HOXB4
HOXB5
HOXB6
HOXB7
HOXB8
HOXB9
HOXB13
mRNA expression
Number corresponding to location along GI-tract

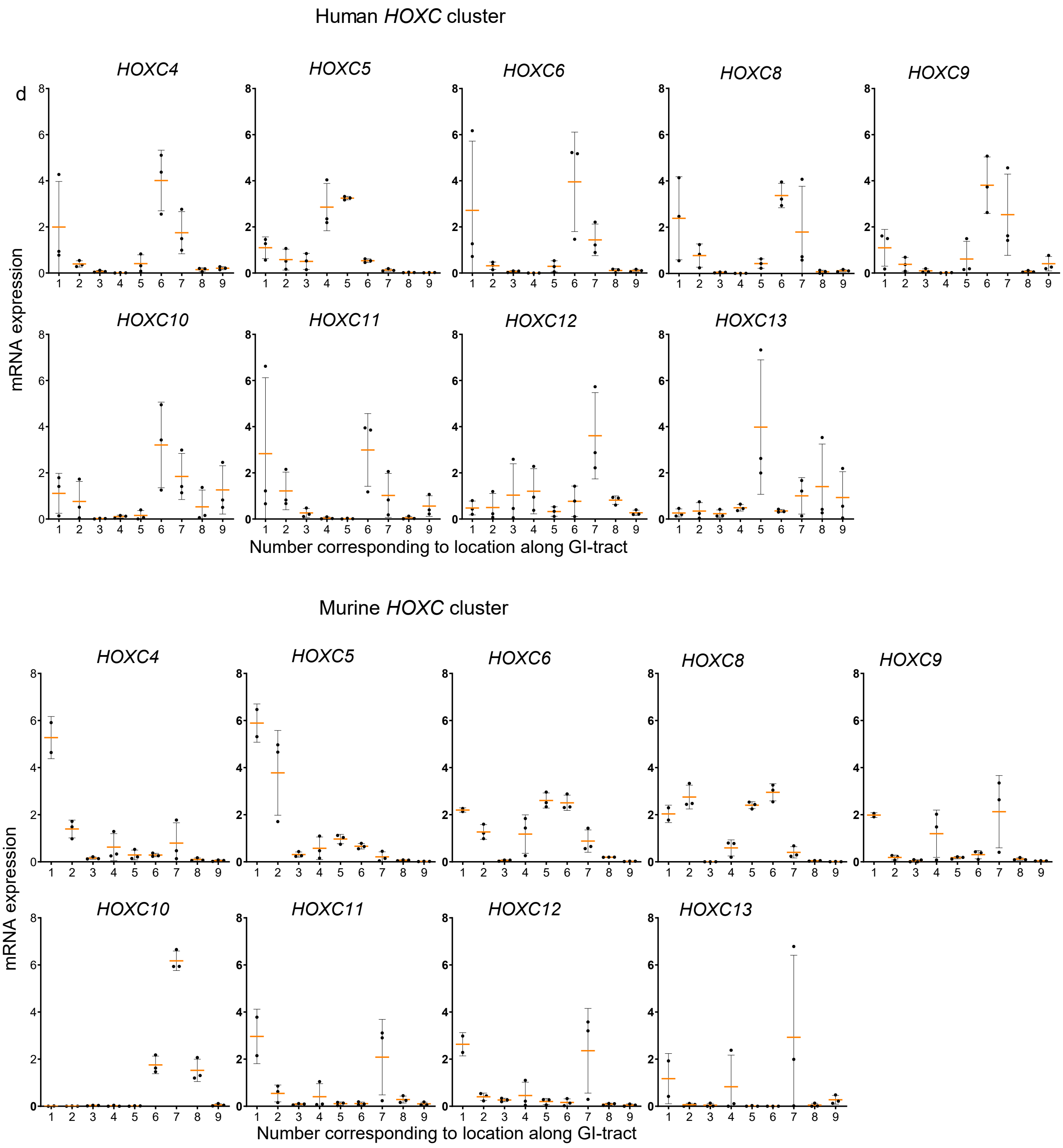
Human *HOXC* cluster
d
HOXC4
HOXC5
HOXC6
HOXC8
HOXC9
HOXC10
HOXC11
HOXC12
HOXC13
mRNA expression
Number corresponding to location along GI-tract
Murine *HOXC* cluster
HOXC4
HOXC5
HOXC6
HOXC8
HOXC9
HOXC10
HOXC11
HOXC12
HOXC13
mRNA expression
Number corresponding to location along GI-tract

Human *HOXD* cluster

e

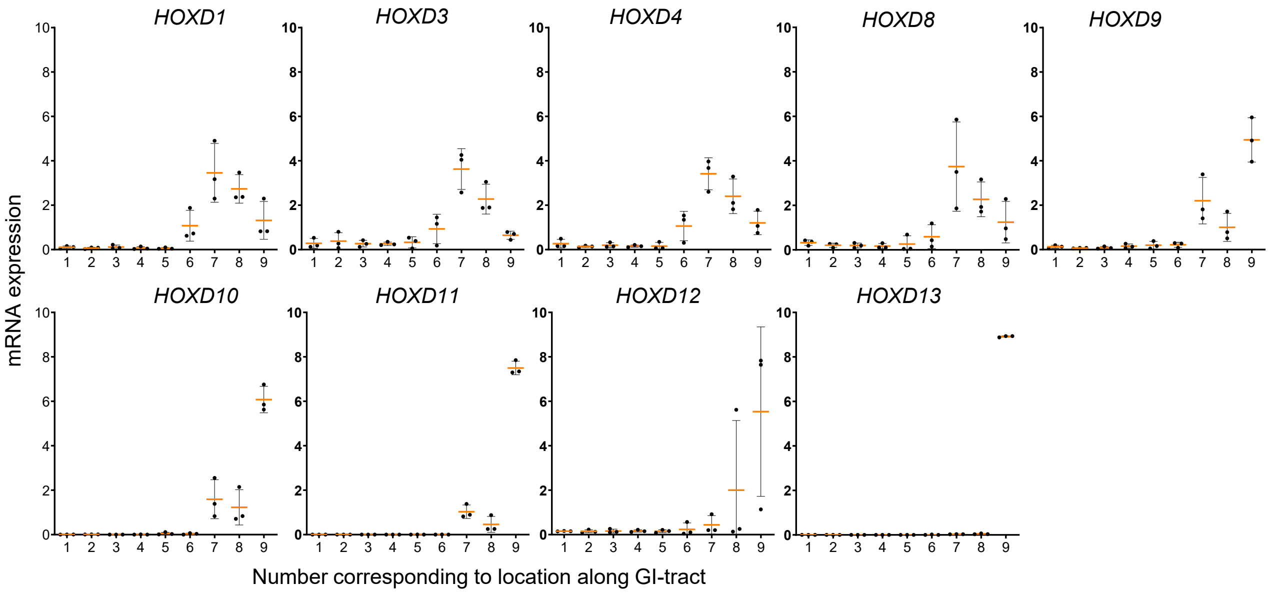

Murine *HOXD* cluster

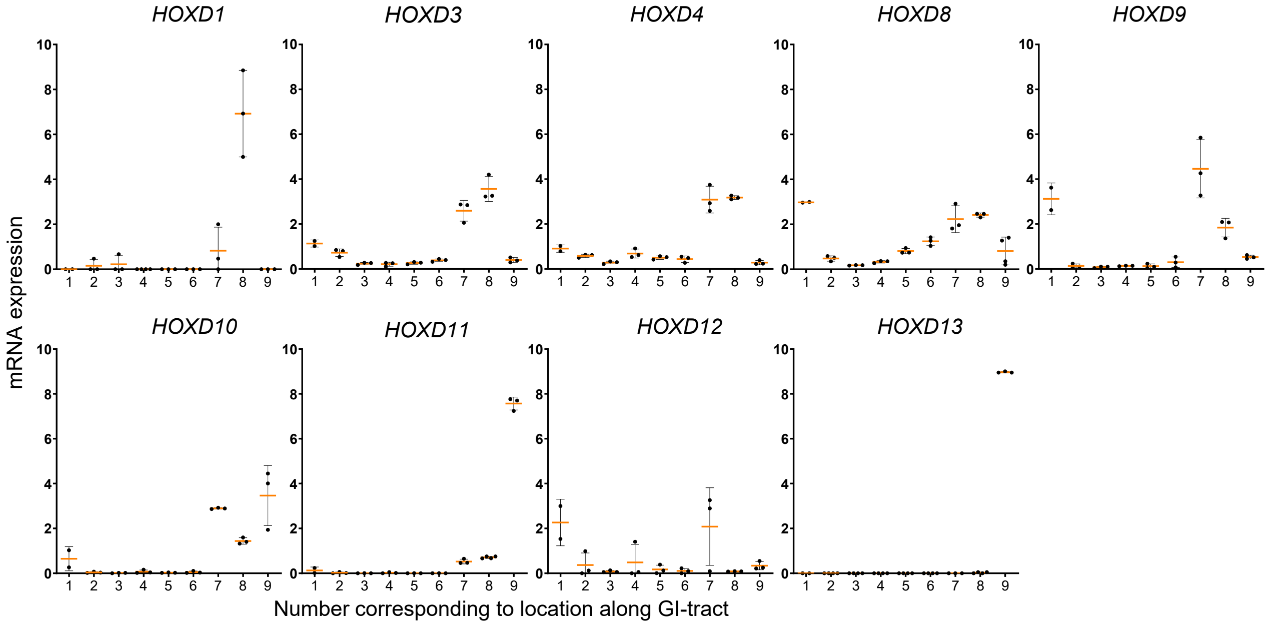

Supplementary fig. 1: *HOX* cluster gene expression along the adult human and mouse gut. a) Overview of *HOX* cluster gene expression in the different epithelial regions along the human and mouse adult GI tract. The Y-axis represents the fold changes of mRNA expression relative to the average mRNA expression of all *HOX* genes of a given cluster. b)-e) Individual *HOX* gene expression of the *HOXA*, *B*, *C*, and *D* clusters is depicted. The Y-axis represents the fold changes of mRNA expression relative to the average mRNA expression of the depicted *HOX* gene. Mean with SD. Human data, n=3 independent samples. Mouse data, n=4 independent samples. GI-gastro-intestinal

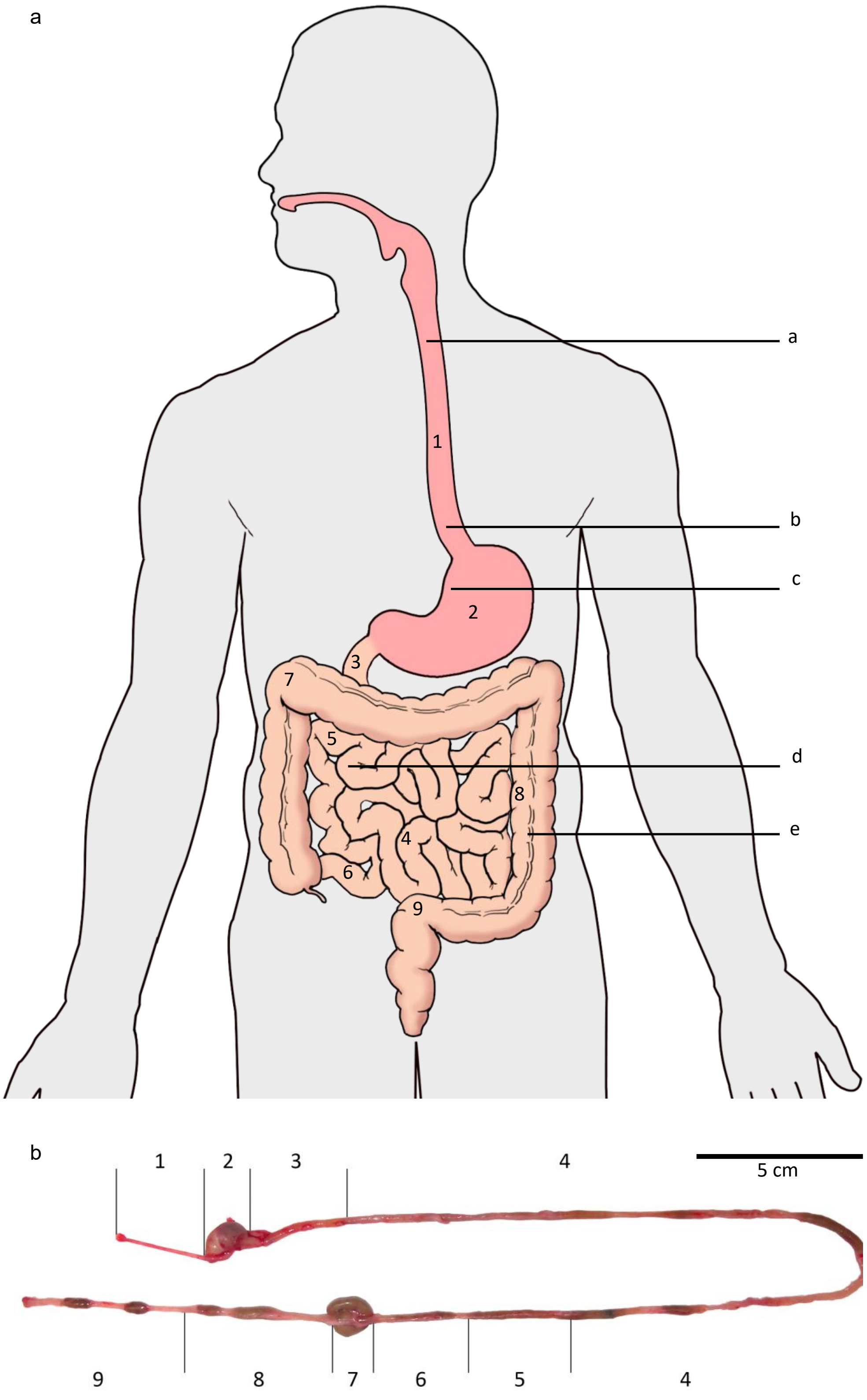

Supplementary fig. 2: locations of forceps biopsies taken along the human GI-tract and sections of murine GI-tract analyzed. a) Location of the forceps biopsies taken along the GI-tract are indicated by number in the schematic illustration of the GI-tract: 1) esophagus, 2) stomach, 3) duodenum, 4) jejunum, 5) proximal ileum, 6) distal ileum, 7) ascending colon, 8) descending colon and 9) sigmoid/rectum. Lesions studied: a) gastric inlet patch; b) columnar-lined esophagus without goblet cells (CLE), Barrett's esophagus (BE), esophageal adenocarcinoma (EAC); c) gastric intestinal metaplasia (IM); d) Meckel’s diverticulum; e) pyloric and Paneth cell metaplasia (from the colon). b) Sections of mouse GI-tract used: 1) esophagus, 2) stomach, 3) duodenum, 4) jejunum, 5) proximal ileum, 6) distal ileum, 7) cecum and proximal colon, 8) proximal colon and distal colon and 9) distal colon.

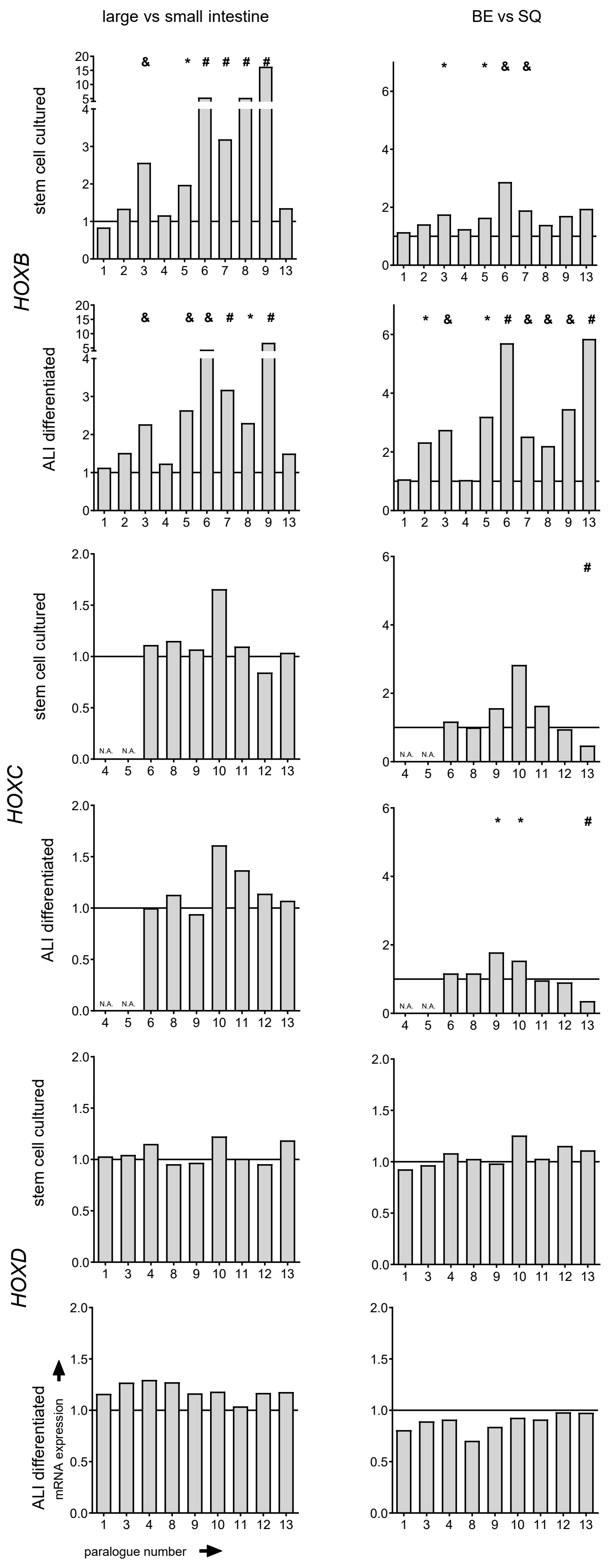
large vs small intestine
BE vs SQ
HOXB
HOXC
HOXD
stem cell cultured
ALI differentiated
N.A.
mRNA expression
paralogue number

Supplementary fig. 3: *HOX* coding is established at the level of the stem cell. Public data of Wang *et al.* [1] contains the mRNA expression of human stem cells isolated from the GI-tract and either cultured as stem cells or differentiated in an air-liquid interface (ALI). Right column: *HOXB*, *C*, and *D* cluster gene expression in the large (n=3 in technical duplicate) compared to the small intestine (n=3 in technical duplicate). Left panels: *HOXB*, *C*, and *D* cluster gene expression in Barret's esophagus (BE, n=12) vs squamous esophagus (SQ) (n=2 in duplicates) in technical duplicates are depicted for stem cell cultures and n=1 each for ALI differentiated samples in technical duplicates. Normalization was performed by setting mRNA expression to 1 for the small intestine or squamous esophagus. *HOX* gene expression in stem cell and ALI cultures are similar, which is not seen in the dataset in general. *HOXB* cluster genes have a higher expression in the large versus the small intestine (left column). No clear regulation of the *HOXC* or *D* clusters is seen, with exception of an upregulation of *HOXC10* in the ascending colon. *HOXB* cluster genes are upregulated in BE stem cells vs squamous esophagus, including mid cluster and 5' *HOXB* genes. *HOXC10* is the most pronounced *HOXC* gene upregulated in BE stem cells. *$p<0.05$; &$p<0.01$; #$p<0.001$; NA: not available. Two-sided t-test was performed. This figure includes no estimate of variance as the empirical Bayes-moderated t-statistic was used which does not generate a standard error.

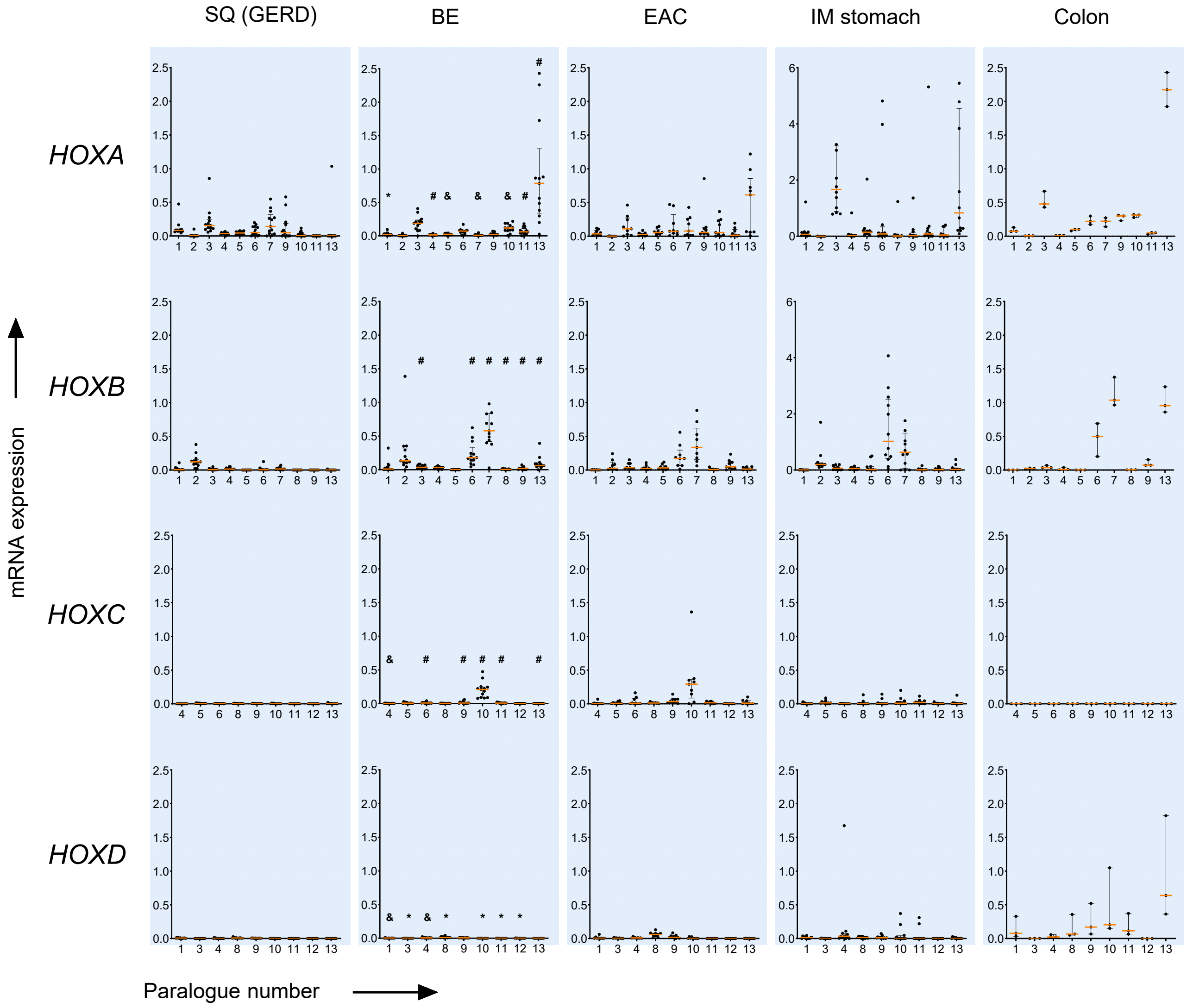

Supplementary fig. 4: *HOX* cluster gene expression in Barrett's esophagus (BE), esophageal adenocarcinoma (EAC), gastric intestinal metaplasia (IM), and colon. Comparison of the mRNA expression levels of the squamous esophagus from GERD (gastro-esophageal reflux disease) patients (n=13), BE (n=13), EAC (n=9), IM of the stomach (n=12), and material from the colon (n=3). Expression in BE was compared to expression in the squamous esophagus (SQ) of matched samples from the same patients (SQ, GERD) by two-sided Student's t-tests. *p<0.05; &p<0.01; #p<0.001. Y-axis values represent the fold change in mRNA expression in relation to the average mRNA expression of all *HOX* genes in all samples. Median with IQR. BE vs Squamous were compared with Wilcoxon matched-pairs signed rank test (two-tailed).

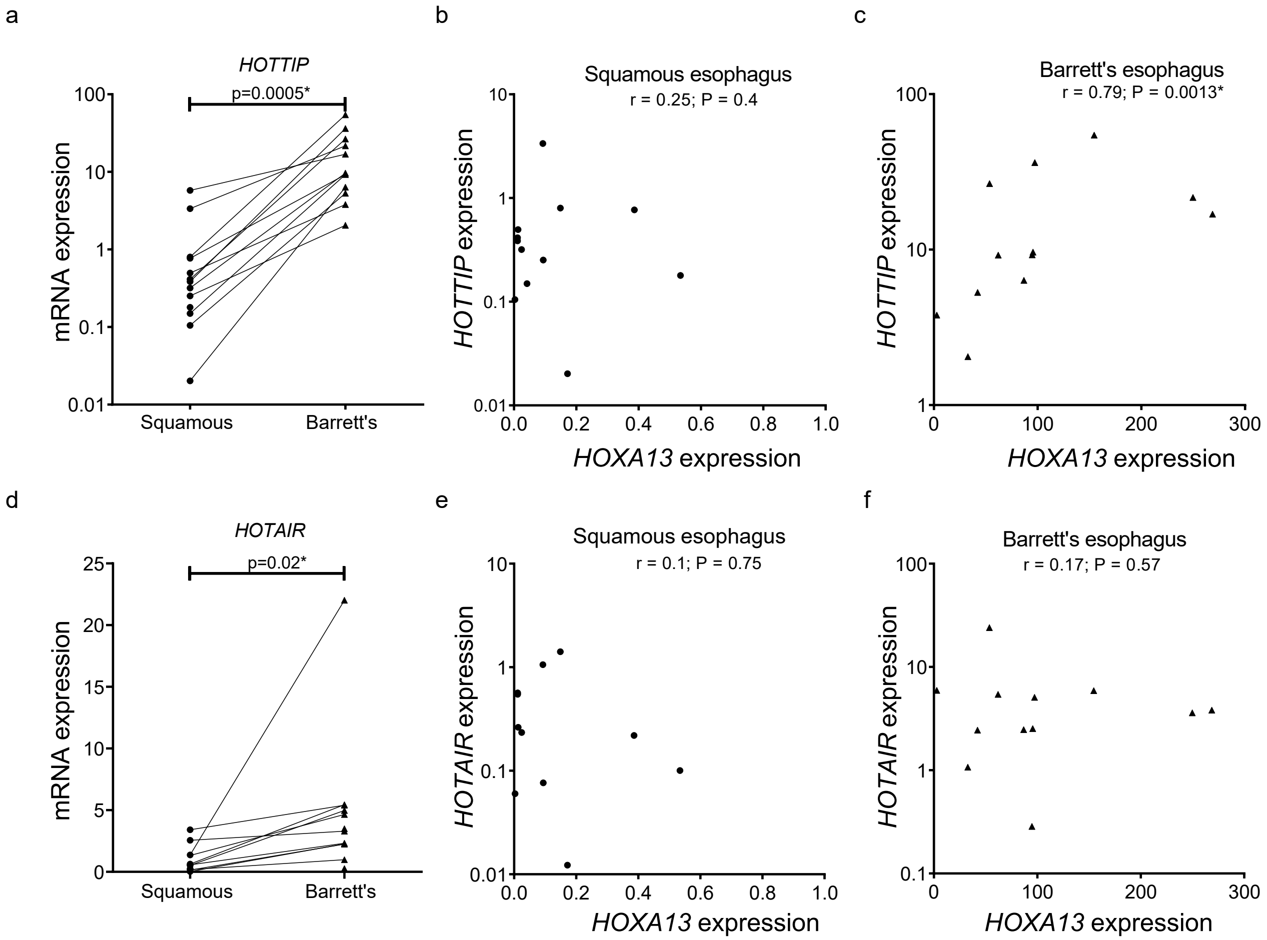

Supplementary fig. 5: Gene expression of lncRNA *HOTTIP* and lncRNA *HOTAIR*, in the normal squamous esophagus and BE tissue from BE patients. a) *HOTTIP* expression, normalized to mean expression in the squamous esophagus, is overexpressed in BE as tested with a Wilcoxon signed rank test. Matched squamous esophageal and BE were taken from the same patient (n=13). b, c) Correlations between expression levels of *HOTTIP* and *HOTAIR* and *HOXA13* in squamous and BE tissues were tested with a non-parametric Spearman test. *HOTTIP* expression does not correlate with *HOXA13* in normal squamous esophagus (b), but does correlate to *HOXA13* in BE tissue (c). d) *HOTAIR* is overexpressed in BE tissue (Wilcoxon signed rank test) and does not correlate with *HOXA13* expression (e, f). *p<0.05; &p<0.01;# p<0.001. Error bars represent the SEM.

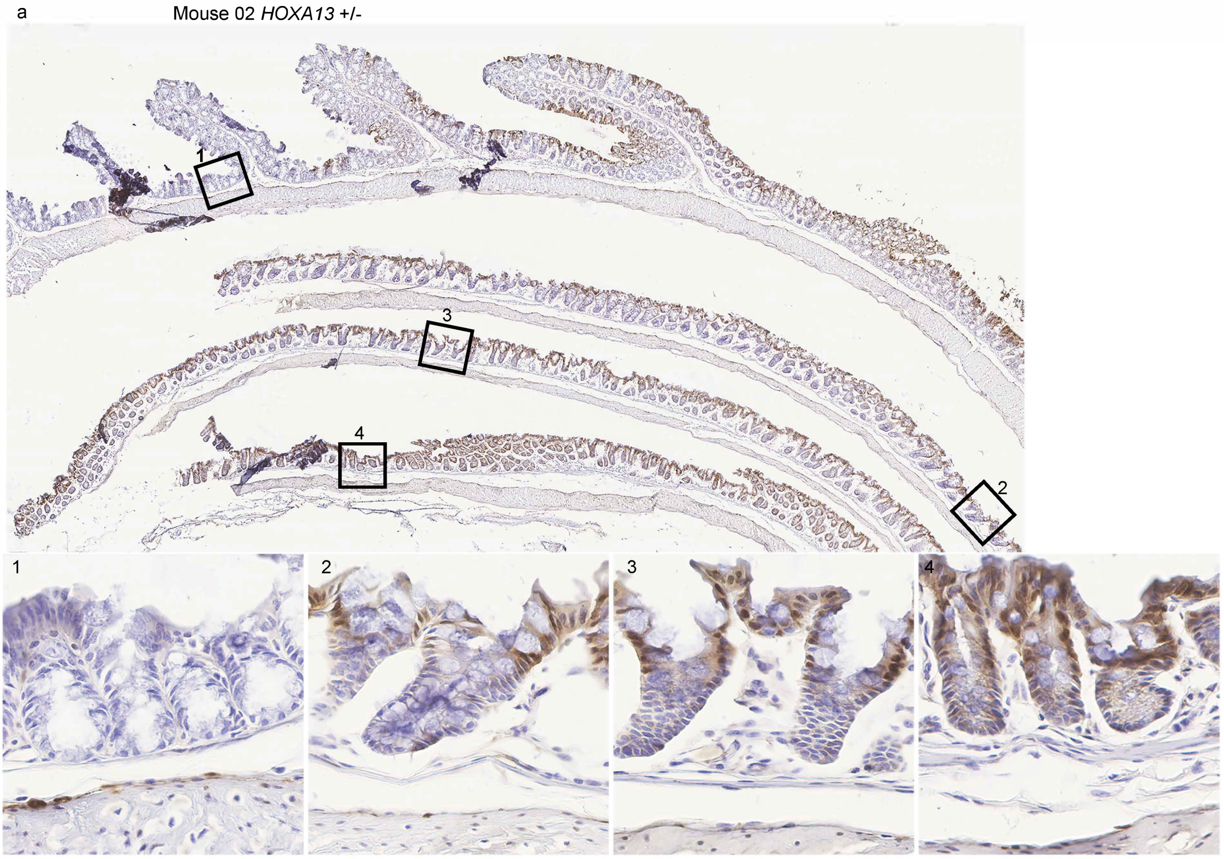
a
Mouse 02 *HOXA13* +/-
1
2
3
4
1
2
3
4

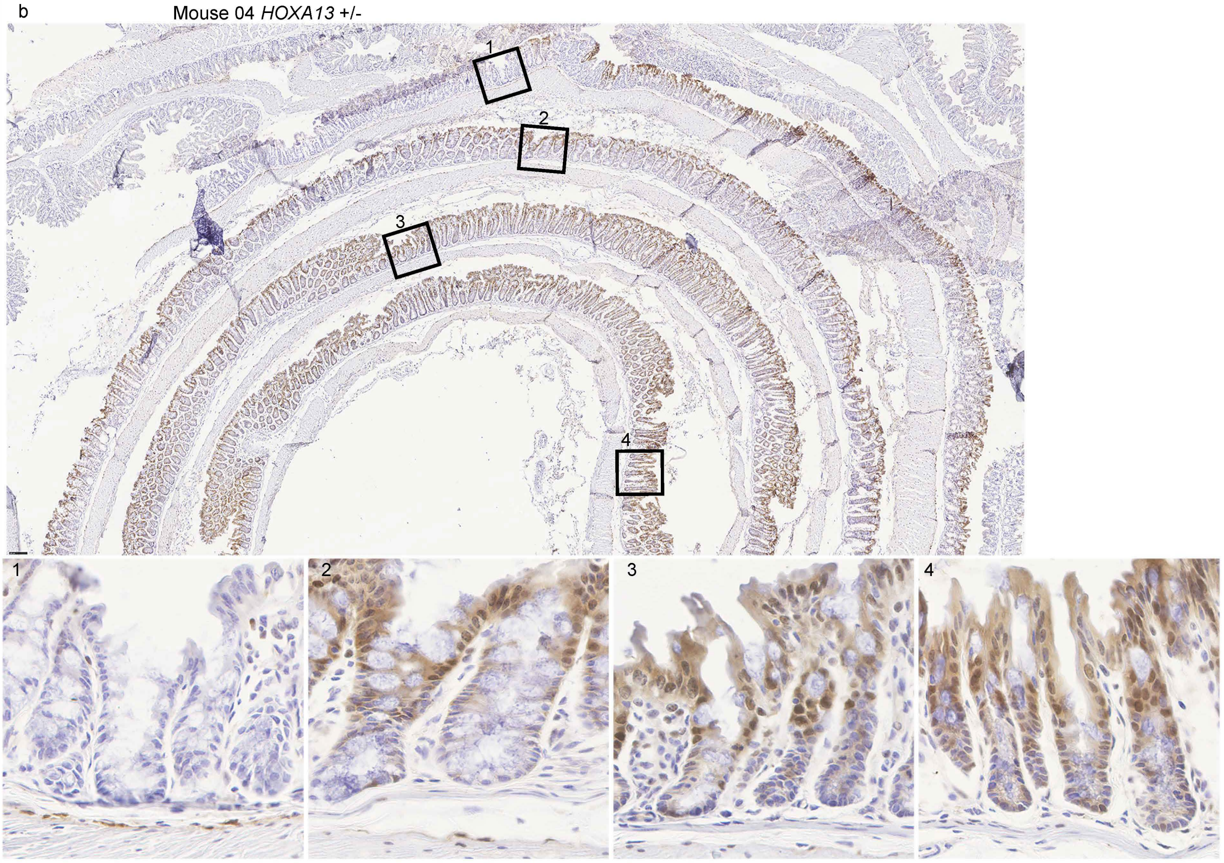
b
Mouse 04 *HOXA13* +/-
1
2
3
4
1
2
3
4

c Mouse 56 *HOXA13* +/-

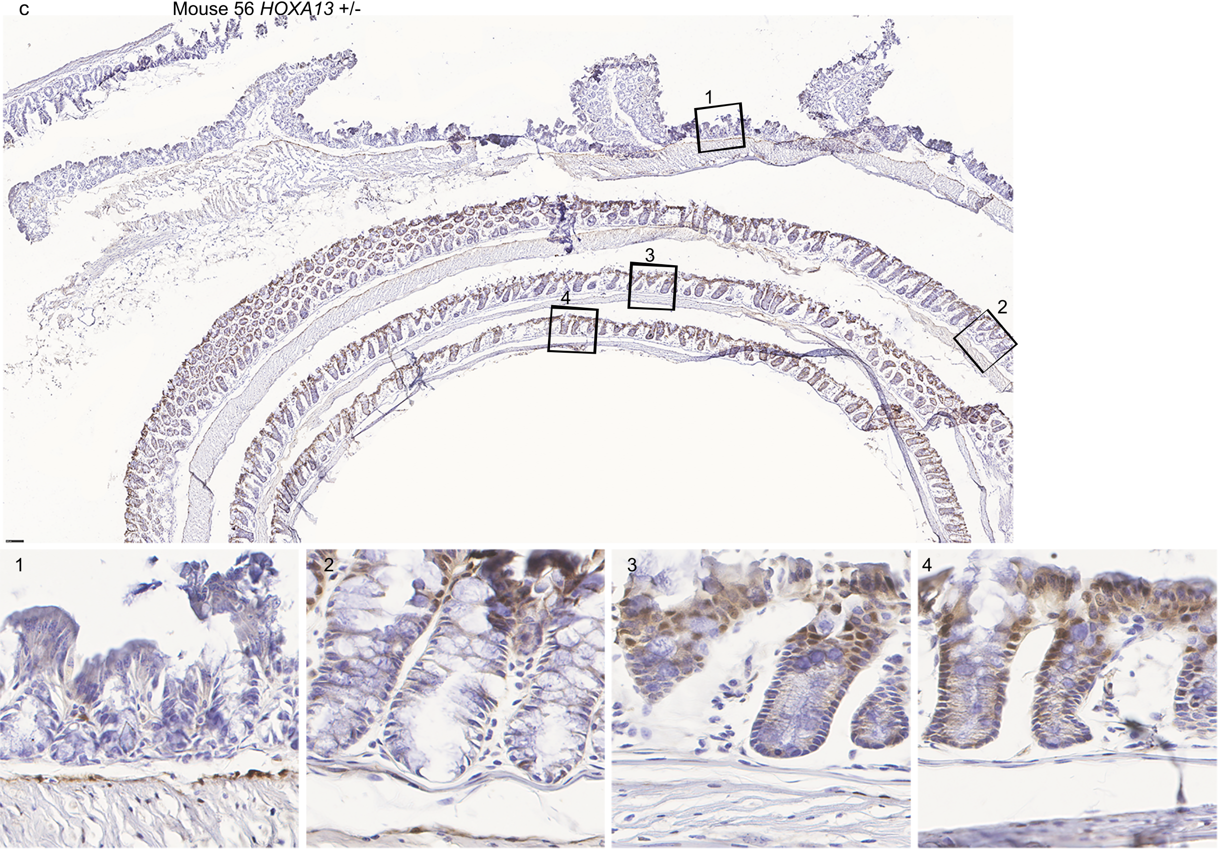

d Mouse 55 *HOXA13* -/-

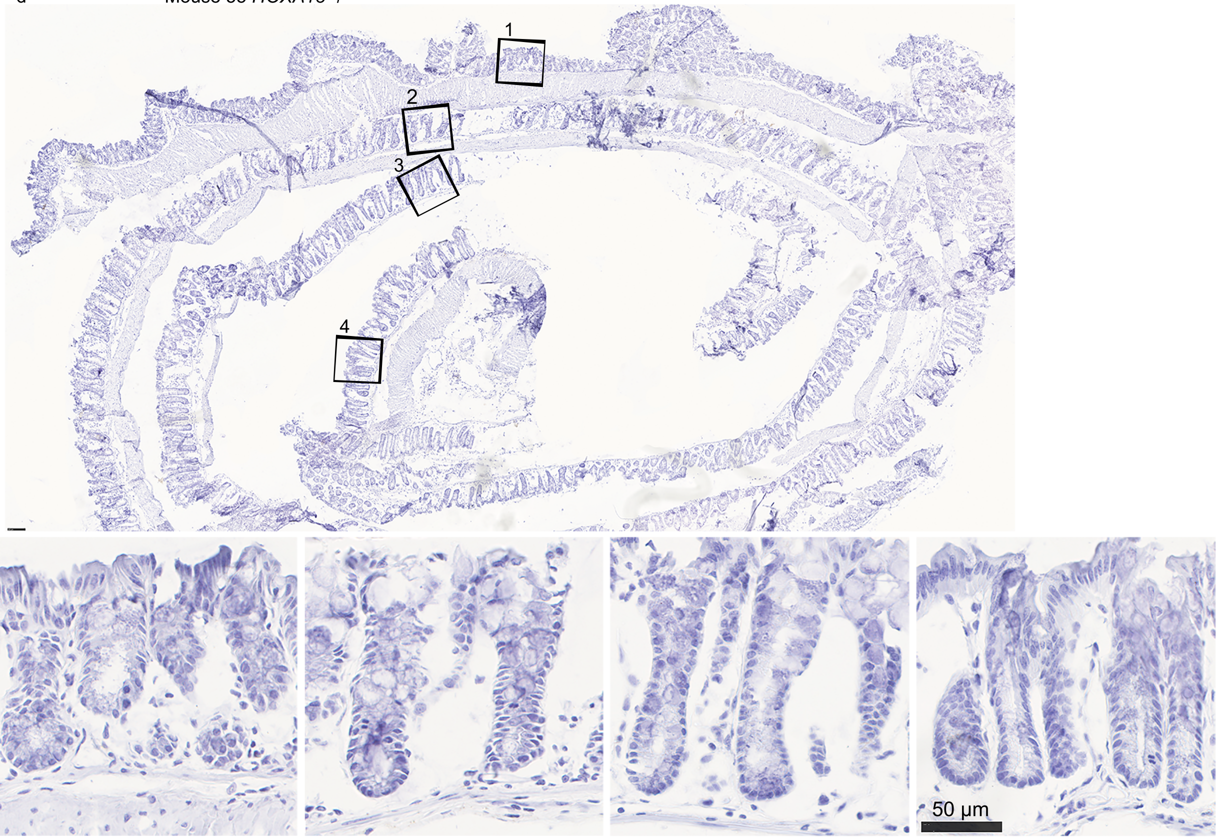

e

Distal expression boundary at anal squamous columnar junction

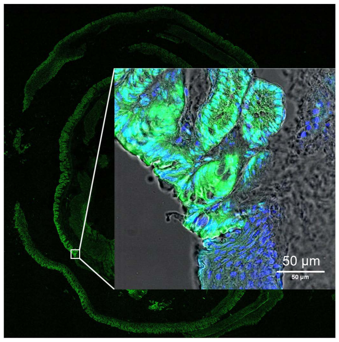

Supplementary fig. 6: Expression of HOXA13 in murine GI tract. Anti-GFP IHC of Hoxa13-GFP mouse model was performed on swiss roles of bowels isolated from three *HOXA13*+/- mice (a-c) and one *HOXA13*-/- negative control mouse (d). Three *HOXA13*+/- mice demonstrated similar result. Overall presentation of swiss role and close ups of proximal (1) and distal (2-4) colon are shown. e) The distal Hoxa13 expression border is the anal SCJ (confocal images).

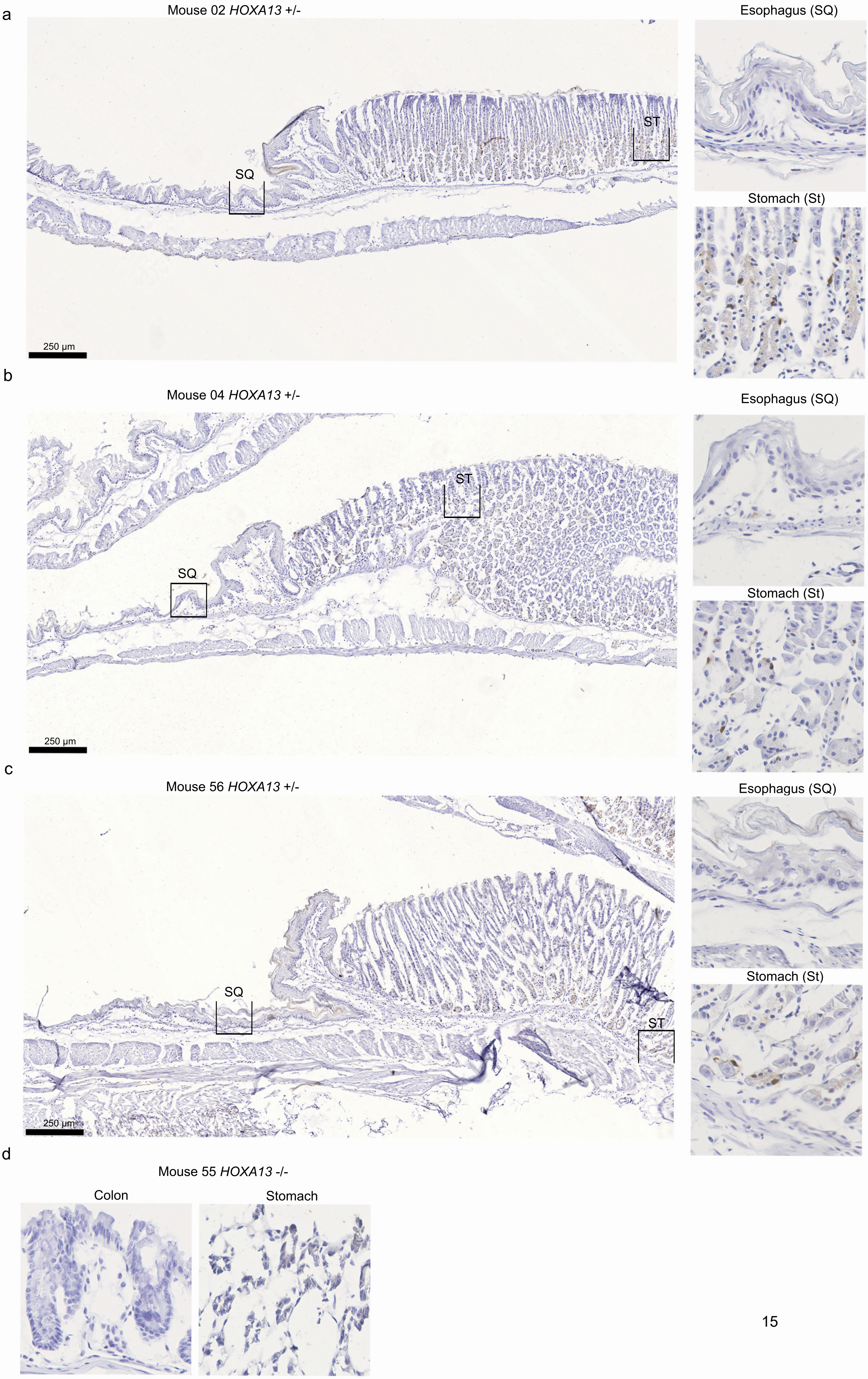

a
Mouse 02 *HOXA13* +/-
SQ
ST
250 µm
Esophagus (SQ)
Stomach (St)
b
Mouse 04 *HOXA13* +/-
SQ
ST
250 µm
Esophagus (SQ)
Stomach (St)
c
Mouse 56 *HOXA13* +/-
SQ
ST
250 µm
Esophagus (SQ)
Stomach (St)
d
Mouse 55 *HOXA13* -/-
Colon
Stomach

Supplementary fig. 7: Expression of HOXA13 in murine upper gastro-intestinal tract. Anti-GFP IHC of Hoxa13-GFP mouse model was performed on upper gastrointestinal tract isolated from three *HOXA13*+/- mice (a-c) and one *HOXA13*-/- negative control mouse (d). Magnification of squamous esophagus and stomach indicated in the overview image are presented on the right. Three *HOXA13*+/- mice demonstrated similar result.

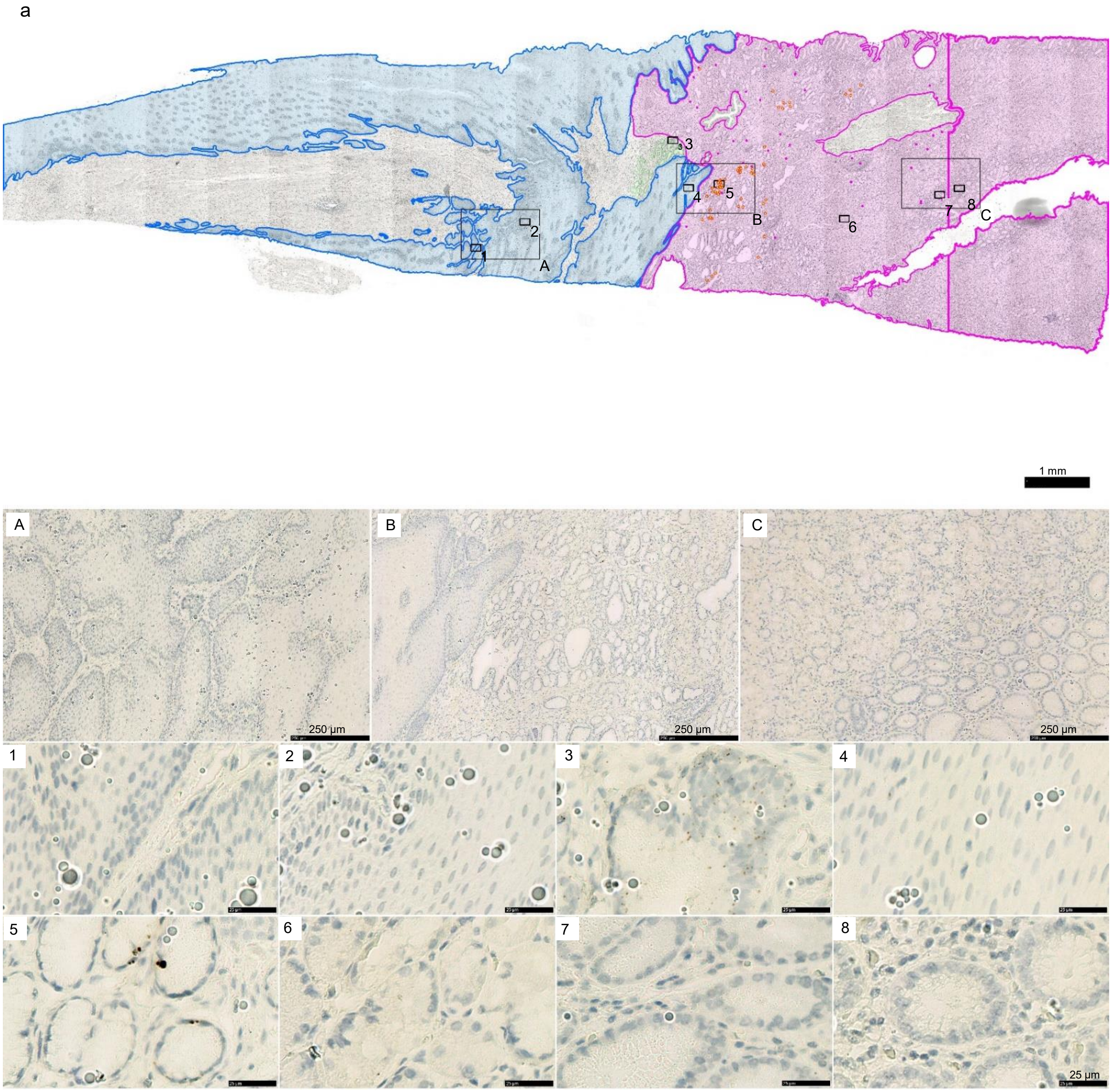
a
3
4
5
B
1
2
A
6
7
8
C
1 mm
A
250 µm
B
250 µm
C
250 µm
1
2
3
4
5
6
7
8
25 µm

++

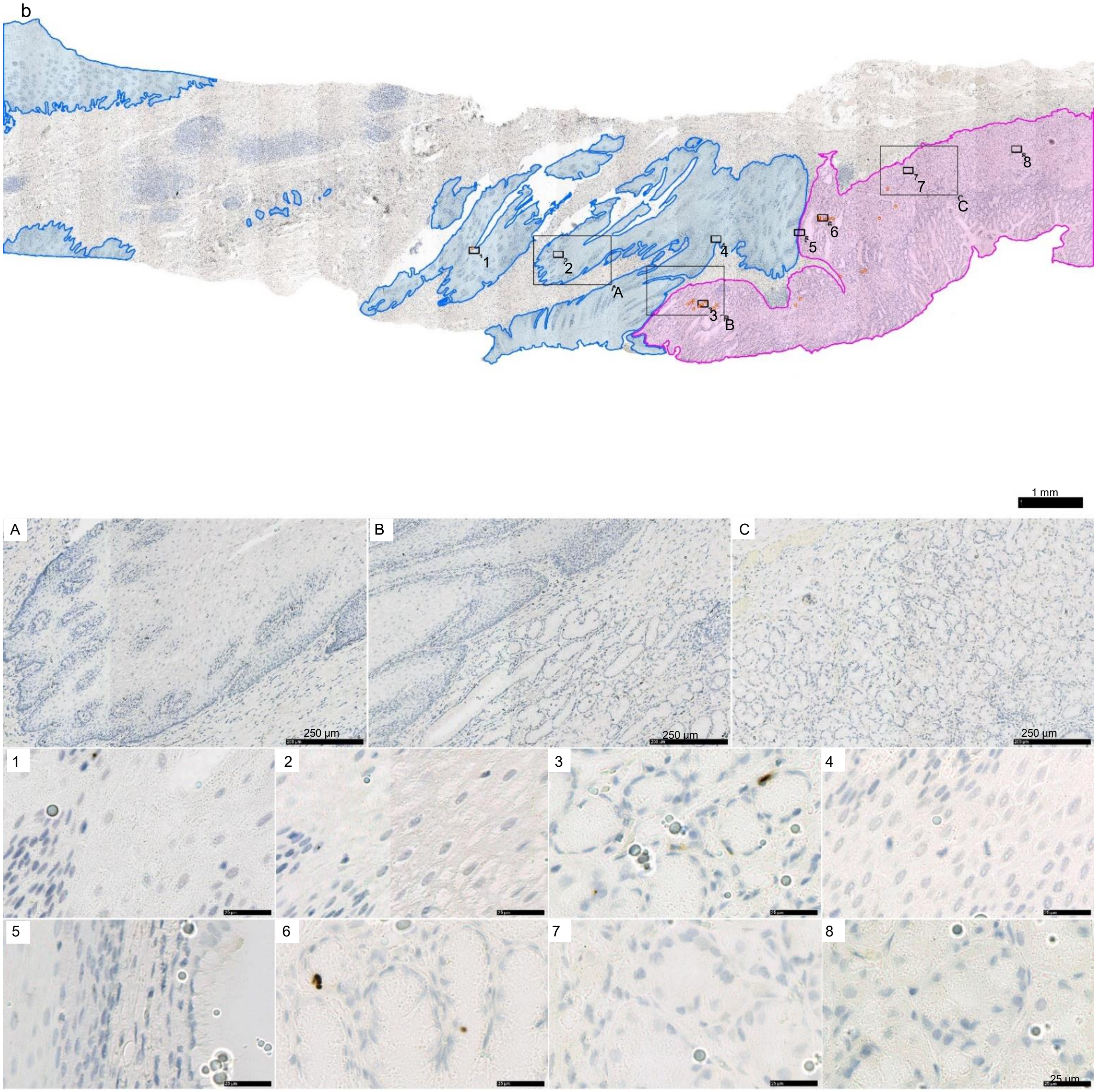
b
1
2
3
4
5
6
7
8
A
B
C
1 mm
250 µm
250 µm
250 µm
25 µm

c

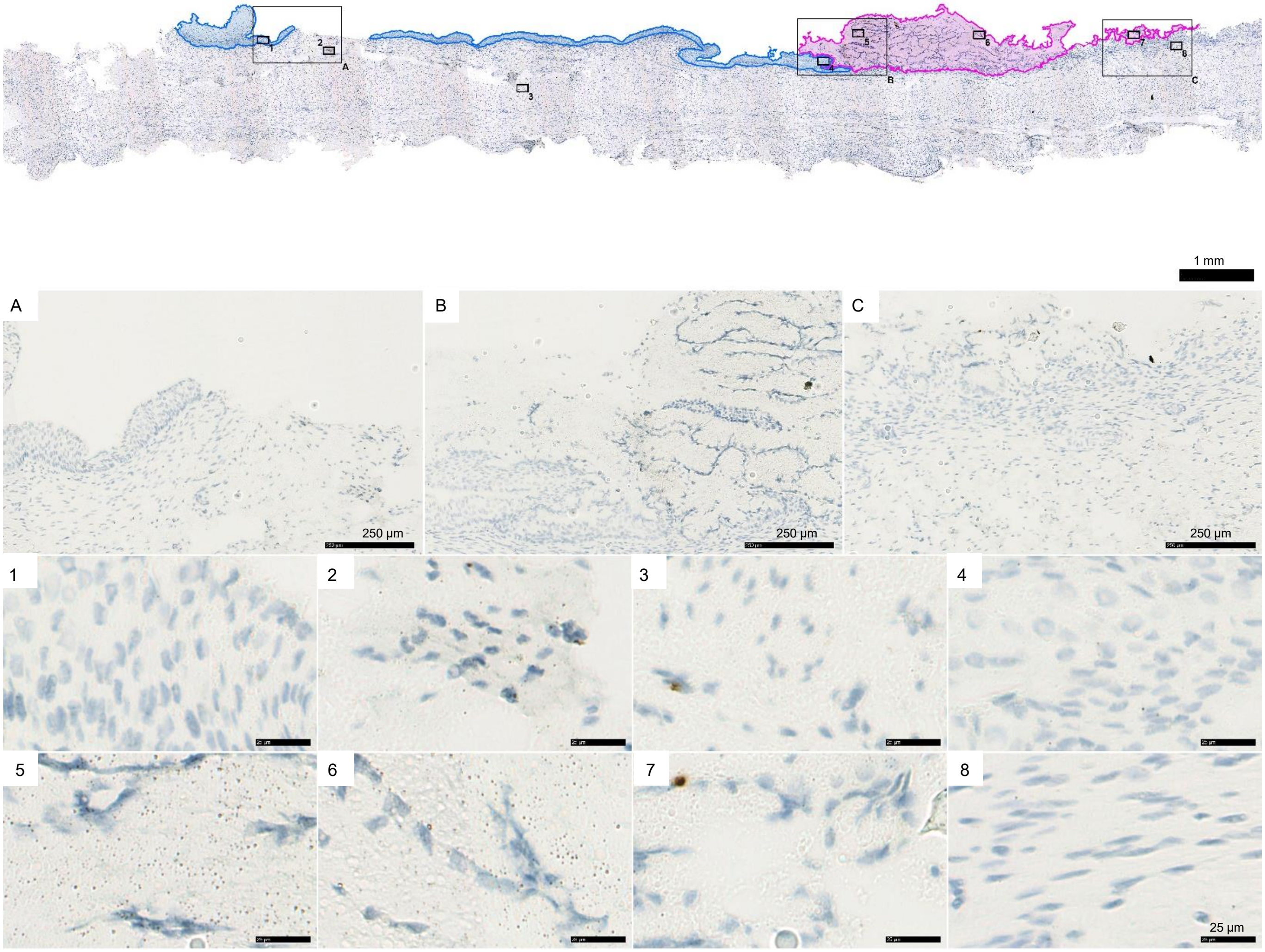

d

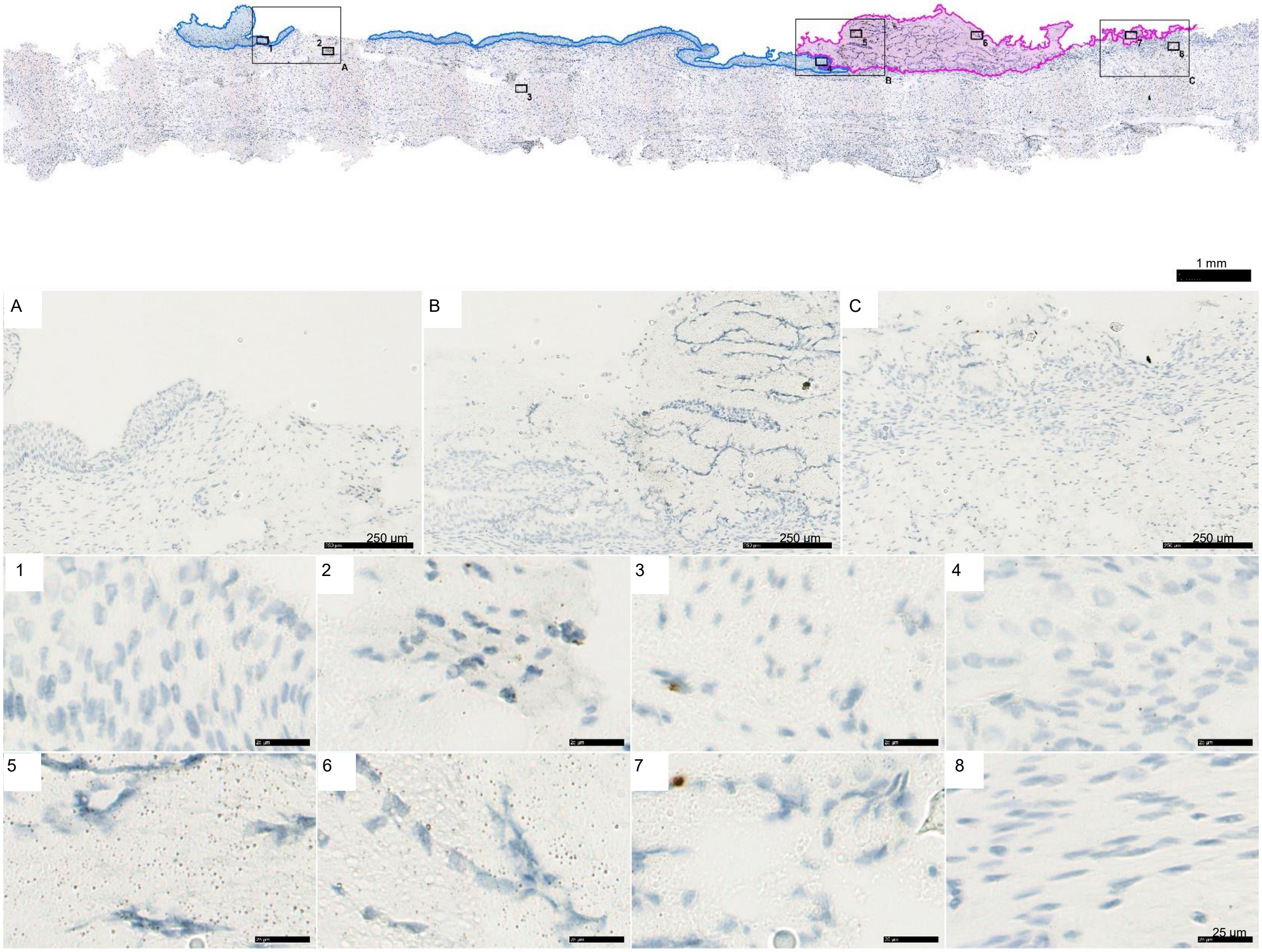

Supplementary fig 8. a, b) *HOXA13* expression as measured by RNA ISH in representative examples of an adult human gastro-esophageal junctions (GEJ) with magnification panel of: A – esophagus, B – GEJ area, C – proximal stomach. n=3 (similar results) c, d) *HOXA13* expression as measured by RNA ISH in a representative examples of an fetus human GEJ with magnification panel of: A – esophagus, B – GEJ area, C – proximal stomach. n=3 (similar results).

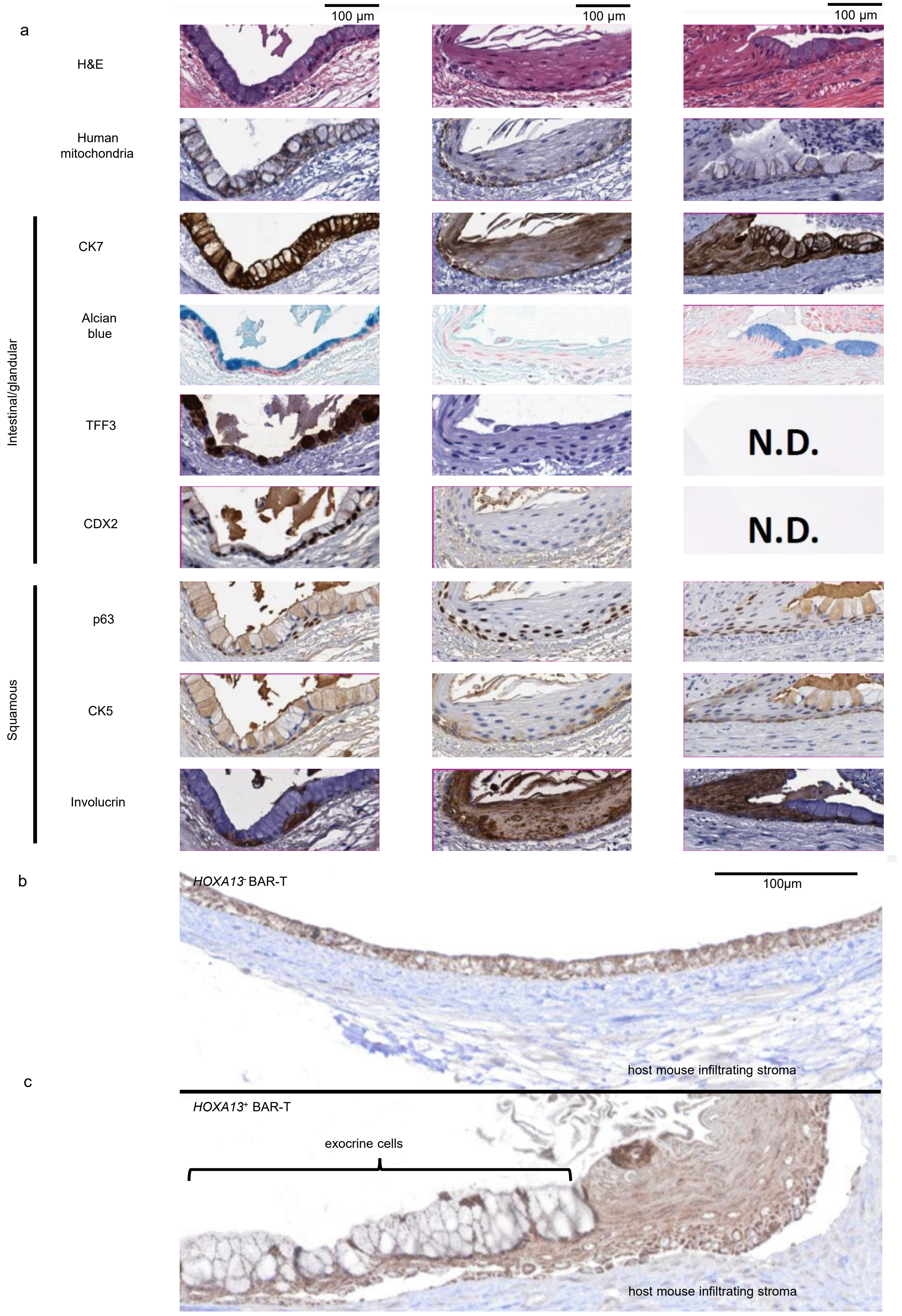
a
100 μm
100 μm
100 μm
H&E
Human mitochondria
Intestinal/glandular
CK7
Alcian blue
TFF3
N.D.
CDX2
N.D.
Squamous
p63
CK5
Involucrin
b
HOXA13⁻ BAR-T
100μm
host mouse infiltrating stroma
c
HOXA13⁺ BAR-T
exocrine cells
host mouse infiltrating stroma

Supplementary fig. 9: Proof of the human origin of epithelium in the rat trachea *in vivo* tissue reconstitution model. a) Staining of the *in vivo* tissue reconstitution model with intestinal/glandular and squamous markers shows that parental BAR-T cells form intestinal-type columnar epithelium (left), squamous epithelium (middle), and multi layered epithelium with mixed phenotype (right) from the same clone. Human origin of the epithelium was confirmed by staining for human mitochondria. b) *HOXA13*$^{-}$ and c) *HOXA13*$^{+}$ representative examples of the BAR-T epithelium stained for human mitochondria. Representative outcomes from 4 independent experiments each with 2-4 replicates. In each experiment and in each replicate we observed the same result of clonal BAR-T cells producing a mixture of the two morphologically distinct epithelia.

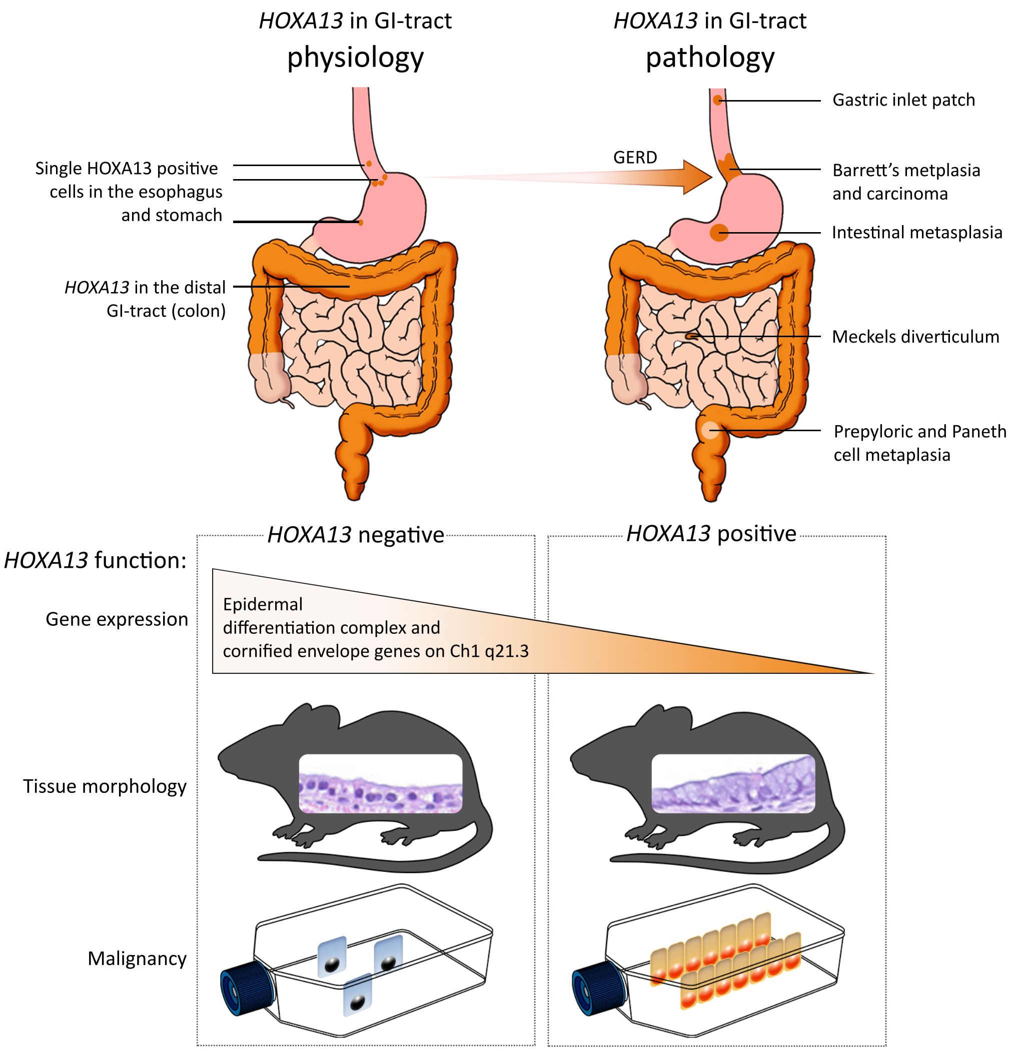

Supplementary fig. 10: summary figure. GI tract - gatro-intestinal tract

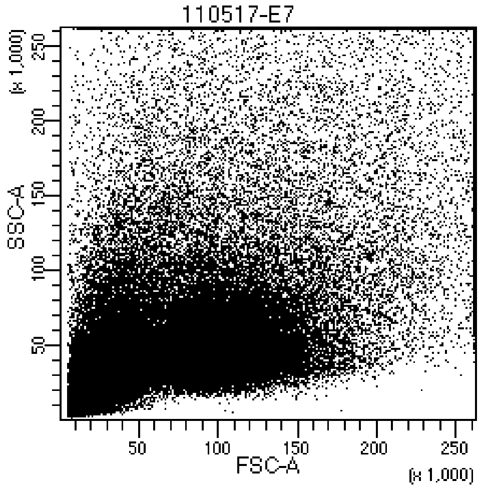

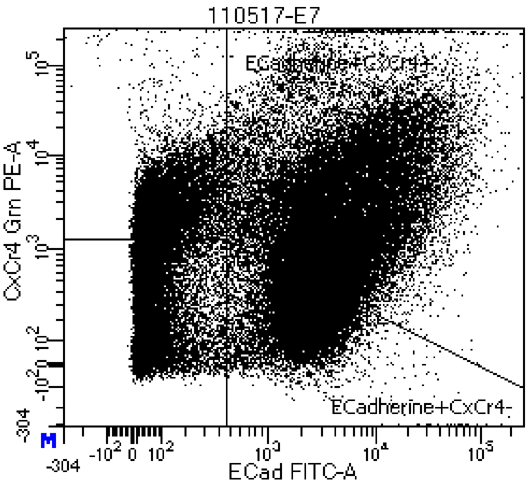

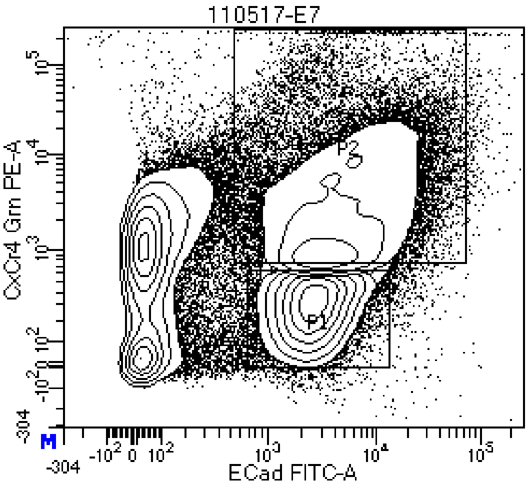

Tube: E7

Population	#Events	%Parent	%Total
All Events	100,000	####	100.0
Q1	13,259	13.3	13.3
ECadherine+CxCr4+	39,401	39.4	39.4
Q3	22,456	22.5	22.5
ECadherine+CxCr4-	24,884	24.9	24.9
P1	30,841	30.8	30.8
P2	29,293	29.3	29.3

Supplementary fig. 11: Panel 1: forward / sideward scatter plot. Panel 2: Dotplot showing cells stained for ECadherin-FITC and CXCR4-PE. Panel 3: contour plots showing gating for ECadherin+ / CXCR4- cells (P1) and ECadherin+ / CXCR4+ (P2) cells, with gates positioned based on negative staining controls.

Supplementary tables

Supplementary table 1. Differentially regulated genes in definitive endoderm versus non-differentiated KH2 mESCs.

	Fold change and q-value of CXCR4$^+$/E-cadherin$^+$ vs non-differentiated mESCs cells		reference
definitive endoderm markers:			
Sox17	12.55	0.00	[2-5]
Foxa1	4.30	0.00	[3, 5]
Gata4	6.86	0.00	[2]
Lgr5	5.39	0.00	[6]
Pax3	25.91	0.00	[3]
Bmp2	16.32	0.00	[4]
Tacstd2	17.55	0.00	[3]
Bmp4	4.73	0.00	[4]
pluripotency markers:			
Nanog	0.20	0.02	[4]
Tcl1	0.01	0.00	[7]
Dppa3	0.02	0.00	[8]

Pluripotent mESC gene expression was compared to CXCR4$^+$/E-cadherin$^+$ FACS selected definitive endoderm cell gene expression. Both samples did not express *HOXA13*. Common definitive endoderm or pluripotency markers are included.

Supplementary table 2. All primers used in this study

primer name	sequence (5' to 3')
huHOXA1 L	TCTTCTCCAGCGCAGACTTT
huHOXA1 R	TTGACCCAGGTAGCCGTACT
huHOXA2 L	CCAAGAAAACCGCACTTCTG
huHOXA2 R	CATCGGCGATTTCCAGG
huHOXA3 L	ATGCAAAAAGCGACCTACTACG
huHOXA3 R	TACGGCTGCTGATTGGCATTA
huHOXA4 L	GAAGAAGATCCATGTCAGCG
huHOXA4 R	GGAACTCCTTCTCCAGCTCC
huHOXA5 L	GCGCAAGCTGCACATAAGTC
huHOXA5 R	GAACTCCTTCTCCAGCTCCA
huHOXA6 L	AAAGCACTCCATGACGAAGG
huHOXA6 R	CATGGCTCCCATACACAGC
huHOXA7 L	CAATTTCCGCATCTACCCCT
huHOXA7 R	GGAACTCCTTCTCCAGCTCC
huHOXA9 L	AATGCTGAGAATGAGAGCGG
huHOXA9 R	GTATAGGGGCACCGCTTTTT
huHOXA10 L	CCGGAGAAGGATTCCCTG
huHOXA10 R	CAGTGTCTGGTGCTTCGTGT
huHOXA11 L	ACACTGAGGACAAGGCCG
huHOXA11 R	GAAGAAGAACTCCCGTTCCA
huHOXA13 L	CCTCTGGAAGTCCACTCTGC
huHOXA13 R	GCACCTTGGTATAAGGCACG
huHOXB1 L	AGGAGACGGAGGCTATTTTCA
huHOXB1 R	GTCTGCTCGTTCCCATAAGGG
huHOXB2 L	CGCCAGGATTCACCTTTCCTT
huHOXB2 R	CCCTGTAGGCTAGGGGAGAG

huHOXB3 L	ATATTCACATCGAGCCCCAG
huHOXB3 R	CGTCATGAATGGGATCTGC
huHOXB4 L	CTTCTCCAGCTCCAAGACCT
huHOXB4 R	CTGGATGCGCAAAGTTCAC
huHOXB5 L	GGAACTCCTTTTCCAGCTCC
huHOXB5 R	GGAAGCTTCACATCAGCCAT
huHOXB6 L	GGGGACATGGACAAAATGAG
huHOXB6 R	GTGAGAACTGAGGAGCGGAC
huHOXB7 L	CTTTCTCCAGCTCCAGGGTC
huHOXB7 R	AACTTCCGGATCTACCCCTG
huHOXB8 L	GAACTCCTTCTCCAGCTCCA
huHOXB8 R	ACACAGCTCTTCCCCTGGAT
huHOXB9 L	TCCAGCGTCTGGTATTTGGT
huHOXB9 R	GAAGCGAGGACAAAGAGAGG
huHOXB13 L	GCTGTACGGAATGCGTTTCT
huHOXC4 L	GAGGTCTGGGGGTTGAGC
huHOXC4 R	GGAGCTGAGACAGGCTCG
huHOXC5 L	GAGTCTGGTAGCGCGTGTAAC
huHOXC5 R	CCACAGATTTACCCGTGGAT
huHOXC6 L	GATCATAGGCGGTGGAATTG
huHOXC6 R	GGCACAGAATGAGGGAAGAC
huHOXC8 L	CAAGGTCTGATACCGGCTGT
huHOXC8 R	ATCAAAACTCGTCTCCCAGC
huHOXC9 L	GTACTTGGTGTAGGGGCAGC
huHOXC9 R	ACAAAGAGGAGAAGGCCGAC
huHOXC10 L	ACCTCTTCTTCCTTCCGCTC
huHOXC10 R	GACACCTCGGATAACGAAGC
huHOXC11 L	ATAAGGGCAGCGCTTCTTG

huHOXC11 R	GAACACAAATCCCAGCTCGT
huHOXC12 L	GCAACTTCGAATAGGGCTTG
huHOXC12 R	AGCTTGGTATCGCCGTTG
huHOXC13 L	GCTGCACCTTAGTGTAGGGC
huHOXC13 R	CCACCTCTGGAAGTCTCCCT
huHOXD1 L	TTCTGTCAGTTGCTTGGTGC
huHOXD1 R	GGATGAAAGTGAAGAGGAATGC
huHOXD3 L	CACCTCCAATGTCTGCTGAA
huHOXD3 R	CAAAATTCAAGAAAACACACACA
huHOXD4 L	AGTTCTAGGACTTGCTGCCG
huHOXD4 R	CTACCCCTGGATGAAGAAGG
huHOXD8 L	TCTTCCTCTTCGTCTACCAGG
huHOXD8 R	TAATATTGGCGAGGACCCAG
huHOXD9 L	CTTTCTCCAGCTCAAGCGTC
huHOXD9 R	CAGCAGCAACTTGACCCAA
huHOXD10 L	TTCTGCCACTCTTTGCAGTG
huHOXD10 R	CTGAGGTCTCCGTGTCCAGT
huHOXD11 L	AAAGAAAAACTCGCGTTCCA
huHOXD11 R	CGAGAAGAGCAGCAGCG
huHOXD12 L	GCTGCTTCGTGTAGGGTTTC
huHOXD12 R	TGAACATGACAGTGCAGGC
huHOXD13 L	CCTCTTCGGTAGACGCACAT
huHOXD13 R	CAGGTGTACTGCACCAAGGA
huRP2 L	AAGCTGAGGATGCTCAAAGG
huRP2 R	CCCATTAAACTCCAAGGCAA
huβ-ACTIN L	GCACAGAGCCTCGCCTT
huβ-ACTIN R	GTTGTCGACGACGAGCG
huGAPDH L	AAGGTCGGAGTCAACGGATTT

huGAPDH R	ACCAGAGTTAAAAGCAGCCCT
moHoxA1 L	AAAAGAAACCCTCCCAAAACA
moHoxA1 R	AGCTCTGTGAGCTGCTTGGT
moHoxA2 L	GATGAAGGAGAAGAAGGCGG
moHoxA2 R	TGCCATCAGCTATTTCCAGG
moHoxA3 L	GCTGCCTGGTCATTCAAAGT
moHoxA3 R	GTCTCCAGTTCCAGGTGCTC
moHoxA4 L	ACCTTGATGGTAGGTGTGGC
moHoxA4 R	ACGCTGTGCCCCAGTATAAG
moHoxA5 L	CTCAGCCCCAGATCTACCC
moHoxA5 R	CAGGGTCTGGTAGCGAGTGT
moHoxA6 L	CCCTGTTTACCCCTGGATG
moHoxA6 R	GTCTGGTAGCGCGTGTAGGT
moHoxA7 L	AAGCCAGTTTCCGCATCTAC
moHoxA7 R	CTTCTCCAGTTCCAGCGTCT
moHoxA9 L	ACAATGCCGAGAATGAGAGC
moHoxA9 R	GTAAGGGCATCGCTTCTTCC
moHoxA10 L	CTCCAGCCCCTTCAGAAAAC
moHoxA10 R	TCTTTGCTGTGAGCCAGTTG
moHoxA11 L	AGGCTCCAGCCTACTGGAAT
moHoxA11 R	CCTTTTCCAAGTCGCAATGT
moHoxA13 L	GCTGCCCTACGGCTACTTC
moHoxA13 R	GCGGTGTCCATGTACTTGTC
moHoxB1 F	GGTGAAGTTTGTGCGGAGAC
moHoxB1 R	TTCGACTGGATGAAGGTCAA
moHoxB2 F	GAACCAGACTTTGACCTGCC
moHoxB2 R	GAGCTGGAGAAGGAGTTCCA
moHoxB3 F	ATCTGTTTGGTGAGGGTGGA

moHoxB3 R	CCGCACCTACCAGTACCACT
moHoxB4 F	GACCTGCTGGCGAGTGTAG
moHoxB4 R	CTGGATGCGCAAAGTTCAC
moHoxB5 F	CTGGTAGCGAGTATAGGCGG
moHoxB5 R	AGGGGCAGACTCCACAGATA
moHoxB6 F	TCCTATTTCGTGAACTCCACCT
moHoxB6 R	GCATAGCCAGACGAGTAGAGC
moHoxB7 F	GAGCAGAGGGACTCGGACTT
moHoxB7 R	GTCTGGTAGCGCGTGTAGGT
moHoxB8 F	CCTGCGCCCCAATTATTATGA
moHoxB8 R	AACTCCTGGATTTGCGAAGGG
moHoxB9 F	TCCAGCGTCTGGTATTTGGT
moHoxB9 R	GAAGCGAGGACAAAGAGAGG
moHoxB13 F	TGCCCCTTGCTATAGGGAAT
moHoxB13 R	ATTCTGGAAAGCAGCGTTTG
moHoxC4 F	CTACCCTGAGCGTCAGTATAGC
moHoxC4 R	CGCAGAGCGACTGTGATTTCT
moHoxC5 F	TTCTCGAGTTCCAGGGTCTG
moHoxC5 R	ATTTACCCGTGGATGACCAA
moHoxC6 F	CAGGGTCTGGTACCGAGAGTA
moHoxC6 R	TCCAGATTTACCCCTGGATG
moHoxC8 F	CAAGGTCTGATACCGGCTGT
moHoxC8 R	ATCAGAACTCGTCTCCCAGC
moHoxC9 F	ACTCGCTCATCTCTCACGACA
moHoxC9 R	GGACGGAAAATCGCTACAGTC
moHoxC10 F	ACCTCTTCTTCCTTCCGCTC
moHoxC10 R	ACTCCAGTCCAGACACCTCG
moHoxC11 F	TCCAACCTCTATCTGCCCAGT

moHoxC11 R	CAAGACGAGTAGCTGTTCCGA
moHoxC12 F	AATACGGCTTGCGCTTCTT
moHoxC12 R	GACCCTGGCTCTCTGGTTTC
moHoxC13 F	GGGCTATGGTTACCCATTTGG
moHoxC13 R	CTGGAGGACAGGTCGTCAC
moHoxD1 F	CAGCACTTTCGAGTGGATGA
moHoxD1 R	GCTCTGTCAGTTGCTTGGTG
moHoxD3 F	ACCAGCTGAGCACTCGTGTA
moHoxD3 R	AGAACAGCTGTGCCACTTCA
moHoxD4 F	CTCCCTGGGCTGAGACTGT
moHoxD4 R	CCCTGGGAACCACTGTTCT
moHoxD8 F	GTAATATTGGCGAGGACCCA
moHoxD8 R	CTACCAGGAGCTTGTGGTC
moHoxD9 F	GCTGAAGGAGGAGGAGAAGC
moHoxD9 R	GTGTAGGGACAGCGCTTTTT
moHoxD10 F	TCTCCTGCACTTCGGGAC
moHoxD10 R	GGAGCCCACTAAAGTCTCCC
moHoxD11 F	GAAAAAGCGCTGTCCCTACA
moHoxD11 R	AGGTTGAGCATCCGAGAGAG
moHoxD12 F	TGCTTTGTGTAGGGTTTCCTCT
moHoxD12 R	CTTCACTGCCCGACGGTA
moHoxD13 F	TGGTGTAAGGCACCCTTTTC
moHoxD13 R	CCCATTTTTGGAAATCATCC
Eef2 F	GCTTCCCTGTTCACCTCTGA
Eef2 R	CGGATGTTGGCTTTCTTGTC
Rpl37 F	GTCGGATGAGGCACCTAAAG
Rpl37 R	GAAGAACTGGATGCTGCGAC
Leng8 F	GGTTGTCTTGAAGCTGCCTT

Leng8 R	GACCTTGGGGTGTAGGGAAT
HOTTIP F	CCTAAAGCCACGCTTCTTTG
HOTTIP R	TGCAGGCTGGAGATCCTACT
HOTAIR F	GGTAGAAAAAGCAACCACGAAGC
HOTAIR R	ACATAAACCTCTGTCTGTGAGTGCC
MEIS1 F	GGGCATGGATGGAGTAGGC
MEIS1 R	GGGTACTGATGCGAGTGCAG
AgeI HoxA13 F	GGTGGTACCGGTGCCACCATGACAGCCTCCGTGCTCCT
XbaI HoxA13 R	ACCACCTCTAGATTAACTAGTGGTTTTCAGTT
HOXA13gibson F	ctccgcggccccgaagccgccaccatggactacaaagacgatgacgacaagATGACAGCCTCCGTG CTC
HOXA13gibson R	cgaagcggccatgaaTTAACTAGTGGTTTTCAGTTTGTTGATG
HOXA13colonyPCR F	CCTCTGGAAGTCCACTCTGC
HOXA13colonyPCR R	GCACCTTGGTATAAGGCACG
pBS31-TetO-F	CCATCCACGCTGTTTTGAC
MF13-R	AGCGGATAACAATTTCACACAGGA
T1E2 HygroR6	TGTATTGACCGATTCCTTGC
T1E2 HygroR7	AGGACATTGTTGGAGCCGAA
PGK-F1	AACAGCTATGACCATG
PGK-F2	GGGCCTTTCGACCTGCATCCATC
Guide1sgRNA F	CACCGTTTCTCTACGACAACGGCGG
Guide1sgRNA R	AAACCCGCCGTTGTCGTAGAGAAAC
px330-F	GATACAAGGCTGTTAGAGAG
TILHOXA13R3	CGAGCAGGGGCTGCATTG
Pre HOXA13 FW2	GCTTTGCATACGCCGTGG
Rat HoxA13 1 F	GGGCTATGACAGCCTCCGT
Rat HoxA13 1 R	ATGTTCTTGTTGAGCTCGTCGG
Rat HoxA13 2 F	GTCGTCTCCCATCCTTCAGA
Rat HoxA13 2 R	TATCCTCCTCCGTTTGTCCTT

Rat HoxA13 3 F	CTGGAACGGCCAAATGTACT
Rat HoxA13 3 R	CCTCCGTTTGTCCTTGGTAA
Rat Hmbs F	TCCTGGCTTTACCATTGGAG
Rat Hmbs R	TGAATTCCAGGTGAGGGAAC
Rat Hprt F	AGGCCAGACTTTGTTGGATT
Rat Hprt R	GCTTTTCCACTTTCGCTGAT
Rat Sdha F	TCCTTCCCACTGTGCATTACAA
Rat Sdha R	CGTACAGACCAGGCACAATCTG
Rat Mapk6 F	TAAAGCCATTGACATGTGGG
Rat Mapk6 R	TCGTGCACAACAGGGATAGA
Rat Rps18 F	AAGTTTCAGCACATCCTGCGAGTA
Rat Rps18 R	TTGGTGAGGTCAATGTCTGCTTTC
HOXA7methF	GACTGCGCCTACCTGAAGAC
HOXA7methR	CAACAGCCCCCTTTATCAGA
HOXA9methF	TGTAGGTCCCCACAGCTACC
HOXA9methR	AATCCTGATTGCCAGCTGAT
HOXA10methF	GGTGTCCTCGTCCCTAGTCA
HOXA10methR	CAGACAGGCAGACACAAGGA
HOXA11methF	TCGAAAAACTGGTCGAAAGC
HOXA11methR	CAATCTGGCCCACTGCTACT
HOXA13methF	AGTACATTTGGCCGTTCCAG
HOXA13methR	CTTCTACCACCAGGGCTACG
HOTTIPmethF	CTTCGAGCGTTTGAAGGAAG
HOTTIPmethR	GTCGCGTTGTGCATTAAGAA

Supplementary table 3. Mouse ESC culture medium and differentiation medium components

Details of used products to culture mESCs.			
Product	Product details		Manufacturer
Dulbecco's Modified Eagle Medium (DMEM)	DMEM 4.5 g/L Glucose with L-Glutamine	82%	Lonza
Foetal Bovine Serum (FBS)		15%	Biowest (Nuaillé, France)
Penicillin/Streptomycin	10,000 Units/mL Penicillin, 10,000 µg/mL Streptomycin	1%	Thermo-Fisher Scientific
MEM Non-Essential Amino Acids		1%	Thermo-Fisher Scientific
Leukaemia Inhibitory Factor (LIF)		0.01%	Department of Developmental Biology, Erasmus MC
2-Mercaptoethanol / β-Mercaptoethanol	55 mM in DPBS	0.1%	Thermo-Fisher Scientific
Sodium Pyruvate	100 mM	1%	Thermo-Fisher Scientific

Details of the components used to differentiate the mESCs to definitive endoderm cells.		
Product	Product details	Manufacturer
Activin A	50 ng/mL	Thermo-Fisher Scientific
(Recombinant Human β-Fibroblast Growth Factor, 154 a.a.)	50 ng/mL	PeproTech EC Ltd. (London, UK)
CHIR	5 µM	Cayman Chemical (Ann Arbor, USA)

Supplementary table 4. All antibodies used in this study.

Details of antibodies.				
Antibody	Concentration	Manufacturer	Product #	RRID
PE rat anti-mouse (clone 2B11) CD184 (CXCR4)	1:250	BD Pharmingen	551966	AB_394305
Alexa Fluor 488 rat anti-mouse (clone DECMA-1) anti-CD324 (E-Cadherin)	1:250	Thermo-Fisher Scientific	53-3249-80	AB_10671270
Anti-human mitochondria (clone 113-1)	1:500	Merck Millipore, Billerica, USA	MAB1273	AB_94052
Mouse anti-human monoclonal (clone OV-TL 12/30) CK7	1:100	Dako Cytomation, Glostrup, Denmark	M7018	AB_2134589
Mouse anti-human monoclonal (clone 415909) TFF3	1:50	R&D Systems, Minneapolis, USA	MAB4407	AB_2271768
Rabbit anti-human monoclonal (clone EPR2764Y) CDX2	1:100	Cell Marque, Rocklin, CA	235R-14	AB_1516797
Mouse anti-human monoclonal (clone DAK-p63) P63	1:100	Dako Cytomation, Glostrup, Denmark	M7317	NA
Rabbit anti-human monocolonal (clone EP1601Y) CK5	1:100	Cell Marque, Rocklin, CA	305R-16	AB_1159468
Rabbit anti-human Involucrin	1:100	gift from A/Prof. Pritinder Kaur, Curtin University, Australia	NA	NA
Mouse anti-human Involucrin	1:500	Sigma-Aldrich, Sigma-Aldrich, St. Louis, Missouri, USA	#I9018	AB_477129
Rabbit polyclonal anti-GFP	1:100	Merck Millipore, Billerica, USA	#AB3080	AB_91337
Rabbit anti-human KRT5 (clone SP27)	0.51 µg/ml	Ventana, USA	760-4935	NA
Rabbit anti-human KR7 (clone SP52	0.536 µg/ml	Ventana, USA	790-4462	NA
Mouse anti-human P63 (cone 4AU)	0.140 µg/ml	Ventana, USA	790-4509	NA

Supplementary method 1. Macro used to quantify *HOXA13*-ISH in FIJI

```
//setTool("freehand");
run("Cut");
run("Internal Clipboard");
selectWindow("Clipboard");
run("Colour Deconvolution", "vectors=[H&E DAB]");
selectWindow("Clipboard-(Colour_2)");
close();
selectWindow("Clipboard-(Colour_1)");
close();
selectWindow("Colour Deconvolution");
close();
selectWindow("Clipboard");
close();
selectWindow("Clipboard-(Colour_3)");
run("Measure");
run("Duplicate...", " ");
selectWindow("Clipboard-(Colour_3)");
setAutoThreshold("Default");
//run("Threshold...");
setThreshold(0, 10);
//setThreshold(0, 10);
run("Convert to Mask");
run("Measure");
close();
selectWindow("Clipboard-(Colour_3)-1");
setThreshold(3, 150);
//setThreshold(3, 150);
run("Convert to Mask");
run("Measure");
close();
String.copyResults();
IJ.deleteRows(0, 4);
```

Supplementary references

1. Wang, X. *et al.* Cloning and variation of ground state intestinal stem cells. *Nature* 522, 173-178 (2015).
2. Li, F. *et al.* Combined Activin A/LiCl/Noggin treatment improves production of mouse embryonic stem cell-derived definitive endoderm cells. *Journal of Cellular Biochemistry* 112, 1022-1034 (2011).
3. Sherwood, R.I. *et al.* Prospective isolation and global gene expression analysis of definitive and visceral endoderm. *Dev Biol* 304, 541-555 (2007).
4. Teo, A.K. *et al.* Activin and BMP4 synergistically promote formation of definitive endoderm in human embryonic stem cells. *Stem Cells* 30, 631-642 (2012).
5. Wang, P. *et al.* A molecular signature for purified definitive endoderm guides differentiation and isolation of endoderm from mouse and human embryonic stem cells. *Stem Cells Dev* 21, 2273-2287 (2012).
6. Tsai, Y.H. *et al.* LGR4 and LGR5 Function Redundantly During Human Endoderm Differentiation. *Cell Mol Gastroenterol Hepatol* 2, 648-662 e648 (2016).
7. Ivanova, N. *et al.* Dissecting self-renewal in stem cells with RNA interference. *Nature* 442, 533-538 (2006).
8. Waghray, A. *et al.* Tbx3 Controls Dppa3 Levels and Exit from Pluripotency toward Mesoderm. *Stem Cell Reports* 5, 97-110 (2015).
9. Polyak, K. & Weinberg, R.A. Transitions between epithelial and mesenchymal states: acquisition of malignant and stem cell traits. *Nat Rev Cancer* 9, 265-273 (2009).

   Shapiro, L. *et al.* Structural basis of cell-cell adhesion by cadherins. *Nature* 374, 327-337 (1995).
