# Supplementary material for: HOXA13 in etiology and oncogenic potential of Barrett’s esophagus
Source: Nat Commun. 2021 Jun 7;12:3354. doi: 10.1038/s41467-021-23641-8 (PMC8184780; doi:10.1038/s41467-021-23641-8)
Supplement: Supplementary file 3 — Description of Additional Supplementary Files [file 41467_2021_23641_MOESM3_ESM.docx]

Description of Additional Supplementary Files

Title: Supplementary Dataset 1.

Description: HOXA13-linked genes in healthy esophagus and in BE

Title: Supplementary Dataset 2.

Description: HOXA13-induced differentially regulated genes in mouse definitive endoderm cells

Title: Supplementary Dataset 3.

Description: HOXA13-induced differentially regulated genes in the BAR-T dataset
