# Supplementary material for: HOXA13 in etiology and oncogenic potential of Barrett’s esophagus
Source: Nat Commun. 2021 Jun 7;12:3354. doi: 10.1038/s41467-021-23641-8 (PMC8184780; doi:10.1038/s41467-021-23641-8)
Supplement: Supplementary file 5 — Supplementary Dataset 2 [file 41467_2021_23641_MOESM5_ESM.zip › 193347_3_data_set_5347016_qfxyfz.docx]

*HOXA13* induced differentially regulated genes in mouse definitive endoderm cells.

| Gene (colon expression relatively high *, low † | Fold Change and q-value of *HOXA13* +/-CXCR4^+^/E-cadherin^+^ DE | | Detailed description |
| --- | --- | --- | --- |
| Epithelial identity: EMT was downregulated (p-value 6,69E-6, z-score -3.184). | | | |
| *Cdh1* | 1.99 | 0.10 | E (epithelial)-cadherin, links the actin cytoskeleton to cell-cell adhesions, inactivation was found to be associated with EMT, leading to diffuse gastric cancer and lobular breast carcinomas ^1^. |
| *Cdh2** | 0.46 | 0.03 | N (neuronal)-cadherin, was found to create cell-adhesion zippers ^2^. |
| *Vim** | 0.43 | 0.10 | Vimentin, from the intermediate filaments type III gene family, was found to be a protein binding to actin associated with the actin core bundle of the brush border ^3^. |
| EMT was downregulated (p-value 6,69E-6, z-score -3.184), i.e. the *HOXA13* overexpressing definitive endoderm cells have a more epithelial identity. | | | |
| *Bmp7*† | 3.49 | 0.00 | Was found to decrease EMT in mouse kidney tubule cells ^4^. |
| *Jag1* | 0.38 | 0.00 | Jagged 1, is a ligand of the NOTCH pathway, this pathway was found to be associated with malignant transformation ^5-7^. |
| *Mgat5** | 4.31 | 0.00 | Mannosyl (alpha-1,6-)-glycoprotein beta-1,6-N-acetyl-glucosaminyltransferase, was found to enhance EMT in mouse keratinocytes ^8^. |
| *Prrx2** | 0.40 | 0.03 | Paired mesoderm homeobox protein 2, was found to promote EMT in breast cancer ^9^. |
| *Scube3* | 0.31 | 0.00 | Signal peptide, CUB domain and EGF like domain containing 3, was found to regulate EMT in lung cancer ^10^. |
| Formation of epidermis” (p-value 3,68E-12, z-score 0,908) was upregulated, in line with the more epithelial character of *HOXA13*+ cells. | | | |
| *Pkp3* | 21.18 | 0.00 | Plakophillin was found to be a desmosomal component ^11^. Was found to mediate desmosome assembly and adherens junction maturation ^12^. |
| *Dsc2* | 2.29 | 0.01 | Desmocollin 2, desmosome component ^13^. |
| *Emp1*† | 1.97 | 0.00 | Epithelial membrane protein 1, was found to be associated with oncogenesis ^14^. |
| *Ppl*† | 2.88 | 0.00 | Periplakin, was found to be localized with ANXA9 in the epidermis ^15^. |
| *Evpl*† | 2.44 | 0.01 | Envoplakin, cornified envelope gene, was found to be downregulated in ESCC ^16^. |
| An upregulation of “actin cytoskeleton signaling” (p-value 3,74E-2, z-score 3,000) was the most pronounced z-score in the pathway analysis of IPA, and directly related to its morphological phenotype ^17^. In line with this is the activation of “integrin signaling” (p-value 1.34E-2, z-score 1,732). | | | |
| *Microvilli:* | | | |
| *Clic5** | 3.51 | 0.00 | Chloride intracellular channel 5, was found to form a complex with EZR and PODXL (FC1.61, *q*=0.16). Was found to be associated with increased migration and invasion in a hepatocellular carcinoma (HCC) cell line together with CLIC5 ^18^. |
| *Ezr** | 2.20 | 0.00 | Ezrin, cytovillin, or villin-2, a cytoplasmic membrane protein and protein-tyrosine kinase substrate in microvilli. It was found to be an intermediate between actin cytoskeleton and plasma membrane. Was found to maintain cell surface structure important for adhesion and migration ^19^. In ESCC it was found to be associated with reduced survival ^20-22^. |
| *Pls3* | 2.55 | 0.00 | Plastin 3, was found to be a microvillus component ^23^. Overexpression increases length and density of microvilli. Abundant Pls3 is associated with cisplatin and UV radiation resistance ^24, 25^. |
| *Vav3** | 2.53 | 0.03 | Vav guanine nucleotide exchange factor 3 is associated with actin cytoskeletal rearrangements. Vav3 was found to be overexpressed in BE and EAC ^26^. |
| *Vill** | 2.58 | 0.048 | Villin like, was found to be associated with and modifies actin filaments in microvilli ^27^. |
| “RhoA signaling” was activated (p-value 1,12E-2, z-score 2,12), although Rhoa itself was not differentially regulated. Villi in RhoA KO mice are noticeably shorter and intestinal epithelial architecture was disorganized compared to that of WT mice ^28^. | | | |
| *Atp8b1** | 2.55 | 0.00 | ATPase phospholipid transporting 8B1, was found to be expressed in differentiated enterocytes and where it was required for apical protein expression and microvillus formation ^29, 30^. |
| *Eps8** | 1.84 | 0.046 | Epidermal growth factor receptor pathway substrate 8, was found to be required for intestinal cell apical morphology ^31^. KO mice have a 25% reduction in intestinal microvilli length ^32^. *Eps8L1* (FC2.98, q=0.02). |
| *Myo5b** | 2.18 | 0.03 | Myosin5B mutations cause microvillus inclusion disease, and disrupt epithelial cell polarity. It is essential for microvillus function ^33^. |
| Keratins: | | | |
| *Krt15*† | 2.50 | 0.054 | Keratin 15, was found to be expressed in the esophagus and glandular prostate cells. Long-lived keratin 15+ esophageal progenitor cells were found to contribute to homeostasis and regeneration ^34^. |
| *KRT19** | 2.34 | 0.00 | Keratin 19, is a columnar keratin ^6^. |
| *KRT20** | 2.84 | 0.01 | Keratin 20, is a more distal GI tract marker ^35^. |
| *Krt81* | 4.01 | 0.00 | Keratin 81, is a hair keratin. It was found to be expressed in normal and breast cancer cells and contributes to their invasiveness ^36^. In PDAC patients receiving chemotherapy it was associated with a dismal prognosis ^37^. It is associated with a malignant phenotype in hepatoblastoma patients ^38^. |
| Tetraspan network: by connecting several molecules, the tetraspan network may organize the positioning of cell surface proteins and play a role in signal transduction, cell adhesion, and motility ^39^. | | | |
| *Cd9* | 2.03 | 0.04 | Motility related protein-1 clustering was found to be accompanied by the formation of microvilli that protrude from either side of adjacent cell surfaces, thus forming structures like micro-villi zippers ^40^. |
| *Cd82*† | 4.12 | 0.00 | CD82 was found to be associated with favorable outcomes in cancer patients ^41^. |
| *Igsf8* | 4.67 | 0.00 | Immunoglobulin superfamily, member 8 links the tetraspanin web to the actin cytoskeleton through their direct association with ezrin-radixin-moesin proteins ^42^. |
| Exocrine function: | | | |
| Molecules from the list “development of exocrine gland” (p-value 8,50E-7, z-score 0,914) in IPA: | | | |
| *Dkk1** | 0.36 | 0.03 | Dickkopf WNT signaling pathway inhibitor 1, was found to decrease differentiation of mucous secretory cells in the mouse small intestine ^43^. |
| *Erbb3* | 2.89 | 0.00 | Human epidermal growth factor receptor 3 was found to limit the number of Paneth cells ^44^. Paneth cell metaplasia in the distal colon occurs exclusively in mucosa affected by inflammatory bowel disease, even in the inactive phase ^45^. This is in line with our observation that Paneth cell metaplasia in the colon is characterized by a decrease in *HOXA13* expression (see main text). As a decrease in *Erbb3* signaling which subsequently does not limit the number of Paneth cells could lead to an increased Paneth cell number. Physiological Paneth cell distribution is inverse to *HOXA13* expression, also in line with the observation in Paneth cell metaplasia. |
| *Fgfr2* | 2.81 | 0.00 | Fibroblast growth factor receptor 2, inhibition of Fgfr2b in rat in organ culture decreases development of pancreas exocrine cells ^46^. |
| *Gcnt3** | 3.51 | 0.00 | Glucosaminyl (N-acetyl) transferase 3, mucin type, was found heavily expressed in colon, small intestine, trachea, and stomach, where mucin is produced ^47^. |
| *Ncoa2* | 1.92 | 0.00 | Steroid receptor coactivator 2 was found critical for progesterone-dependent uterine function and mammary morphogenesis in the mouse ^48^. |
| Shh signaling: | | | |
| *Ptch1** | 2.26 | 0.02 | Patched 1, its levels correlate with Hedgehog pathway activity ^49^. It was found increased in BE, and may contribute to its etiology ^50^. |
| *Smo* | 0.62 | 0.04 | Smoothened, frizzled class receptor, was found to decrease SMO expression correlates with less Hedgehog pathway activity. Hedgehog pathway activity serves to induce the proximal tractus, especially the esophagus ^51^. |
| Wnt signaling: | | | |
| *Dkk1* | 0.36 | 0.03 | Dickkopf WNT signaling pathway inhibitor 1, see above. |
| *Fzd7* | 2.10 | 0.03 | Frizzled class receptor 7, deletion in mouse adult intestinal epithelium was found to lead to stem cell loss in vivo and organoid death in vitro ^52^. |
| *Wnt2b* | 0.23 | 0.00 | Wnt family member 2b. |
| *Wnt3** | 0.47 | 0.053 | Wnt family member 3 was found to be necessary and sufficient to induce Paneth cell formation ^53^. *Wnt3* is produced specifically by Paneth cells ^54^. |
| *Wnt5a** | 0.37 | 0.03 | Wnt family member 5a, overexpression from 10.5 dpc until 18.5 dpc was found to result in drastic shortening of the small intestine, colon, cecum and stomach ^55^. It inhibits proliferation of intestinal epithelial stem cells ^56^. It up-regulates the expression of the tumor suppressor 15-PGDH and induces differentiation of colon cancer cells ^57^. |
| *Wnt10a* | 3.21 | 0.00 | Wnt family member 10A was found to promote an invasive and self-renewing phenotype in ESCC ^58^. |
| Sox signaling: | | | |
| *Sox9** | 2.55 | 0.00 | SRY-box 9, was found to be correlated with ERBB3 in pancreatic ductal AC ^59^. Sox9 induces Paneth cells which is not in line with the function of Erbb3 and Wnt3 ^60^. SOX9 is essential in the development of embryonic CLE, but is switched off in post-natal life, and is re-expressed again in BE ^61-64^. Sox9 was found to drive columnar differentiation of SQ ^65^. |
| *Sox11** | 0.60 | 0.00 | SRY-box 11, knock out was found to cause hypoplasia of the lung, stomach, and pancreas ^66^. |
| *Sox13* | 3.03 | 0.00 | SRY-box 13, expressed in pancreas and liver, an autoantigen in primary biliary cirrhosis, Sox13 was found to inhibit canonical Wnt signaling in T cells ^67^. |
| Glypicans: | | | |
| *Gpc1* | 2.41 | 0.00 | Glypican 1, can be a biomarker for relapse of stage III CRC and may be involved in EMT activation, invasion, and migration of CRC ^68^. It was found to be a marker of pancreatic cancer and ex vivo it appears to have an oncogenic role ^69^. GPC1 is an independent prognostic factor in ESCC ^70^. |
| *Gpc2* | 0.51 | 0.049 | Glypican 2, a neuroblastoma oncoprotein and candidate immunotherapeutic target ^71^. |
| *Gpc3* | 0.23 | 0.00 | Glypican 3, is a fetal gut marker ^72^. |
| *Gpc6* | 0.24 | 0.00 | Glypican 6, was found to be hypermethylated in cancer and mRNA is down-regulated ^73^. |
| Interleukin: | | | |
| *Il1rn* | 5.15 | 0.00 | Interleukin 1 receptor antagonist, was found to be downregulated in BE and EAC ^74^ typical SQ marker, downregulated in ESCC ^75^. |
| *Il5ra* | 3.06 | 0.00 | Interleukin 5 receptor subunit α, heterozygous mutants were more frequent in atopic dermatitis of Koreans ^76^, which implicates il5ra in epithelial homeostasis. |
| *Il22ra1* | 3.10 | 0.01 | Interleukin 22 receptor subunit α 1, was found to mediate fucosylation promotes intestinal colonization resistance to E. faecalis ^77^. It was found to have a higher expression in the colon compared to the cecum in line with the expression of HOXA13 in the colon with exception of the cecum ^78^. |
| Apoptosis/necrosis: | | | |
| *Casp8** | 2.30 | 0.00 | Caspase 8, high Caspase-8 was found to be a negative markers of OS ^79^. |
| *Cxcl1** | 3.15 | 0.00 | CXC motif chemokine ligand 1, was found important for mucosal barrier ^80^. |
| *Ripk3* | 2.19 | 0.04 | Receptor interacting serine/threonine kinase 3, a substrate of caspase 8 ^81, 82^. The RIPK1-RIPK3 necrosome was found to promote pancreatic oncogenesis through upregulation of the chemokine CXCL1 (See below)^83^. Loss of RIPK3 appears to promote tumors in the colon ^84^. |
| BE/metaplasia specific, oncogenic molecules: | | | |
| *Anxa1*† | 1.69 | 0.00 | Annexin A1, was found to promote the proliferation of ESCC cells ^85^. High expression is frequent in EAC and GEJ AC, correlates with more advanced pathologic T stage and the presence of distant metastasis, and is an independent prognostic factor for patient survival ^86^. |
| *Artn* | 2.21 | 0.04 | Artemin, is hypoxia responsive and was found to promote oncogenicity and increased tumor initiating capacity in HCC ^87^. |
| *Fgfr2* | 2.81 | 0.00 | Fibroblast growth factor receptor 2, has been found to be highly expressed early in progression from BE to EAC *^88^.* |
| *Fgf13* | 2.37 | 0.02 | Fibroblast growth factor 13, tumor suppression by TP53 was found to be involved in inhibiting *FGF13* ^89^. |
| *Sparc* | 0.41 | 0.00 | Secreted protein acidic and cysteine rich, expression correlates with matrix metallopeptidase 2 (*MMP2*; FC=0.25, *q*=0.00) expression in esophageal tumors, and high SPARC expression was found to be correlated significantly with lymph node metastasis and poor patient prognosis ^90^. |
| Oncogenic molecules: | | | |
| *Atp2c2** | 7.38 | 0.00 | ATPase secretory pathway Ca^2+^ transporting 2, the Orai1-Atp2c2 complex was found to be associated with constitutive store-independent Ca^2+^ signaling which promotes tumorigenesis ^91^. |
| *Cd55* | 4.79 | 0.00 | Decay accelerating factor, was found to prohibits formation of the membrane attack complex indirectly ^92^. |
| *Ezr** | 2.20 | 0.00 | Ezrin, was found to be associated with reduced survival in ESCC ^20-22^. |
| *Nek2** | 2.35 | 0.01 | NIMA related kinase 2, was found to be overexpressed in a wide variety of human cancers implicating in various aspects of malignant transformation, including tumorigenesis, drug resistance and tumor progression. NEK2 inhibitors are being developed ^93^. |
| *Nkd1** | 0.22 | 0.00 | Naked cuticle homolog 1, downregulation was found to promote HCC progression and promotes gastric cancer migration and invasion ^94, 95^. *NKD1* expression is reduced in HCC and is associated with a poor prognosis ^96^. |
| *Pim1** | 2.61 | 0.00 | Pim-1 proto-oncogene, serine/threonine kinase, was found to regulate glycolysis and promotes tumor progression in HCC ^97^. Pim1 is a pro-survival kinases that is commonly amplified in cancer ^98^. |
| *Slc2a1* | 2.01 | 0.01 | Solute carrier family 2 member 1, was found to be a marker of HGD, EAC and gastric cancer ^99, 100^. *Slc2a1* expression in CRC is independently associated with poor prognosis ^101^. |
| *Spink6* | 15.13 | 0.00 | Serine peptidase inhibitor, Kazal type 6, in nasopharyngeal carcinoma was found to promote metastasis ^102^. |

Molecules associated with epithelial identity, the cytoskeleton (microvilli, keratins, and the tetraspan network), exocrine function, oncogenic molecules, and specific signaling pathways were included. All fold change and *q* values depicted pertain to the BAR-T dataset. Information in the “known function” and “Detailed description” columns was obtained through non-systematic review and should not be considered as an exhaustive overview of the literature. In the first column, molecules of which the direction of regulation by *HOXA13* is in line with their relative expression in the distal compared to the proximal GI-tract are indicated with an *, the reverse with a †. If no clear relationship was present no symbol was added to the gene name.

Reference list:

1. Polyak, K. & Weinberg, R.A. Transitions between epithelial and mesenchymal states: acquisition of malignant and stem cell traits. *Nat Rev Cancer* 9, 265-273 (2009).

2. Shapiro, L. *et al.* Structural basis of cell-cell adhesion by cadherins. *Nature* 374, 327-337 (1995).

3. Friederich, E., Vancompernolle, K., Louvard, D. & Vandekerckhove, J. Villin function in the organization of the actin cytoskeleton. Correlation of in vivo effects to its biochemical activities in vitro. *J Biol Chem* 274, 26751-26760 (1999).

4. Zeisberg, M. *et al.* BMP-7 counteracts TGF-beta1-induced epithelial-to-mesenchymal transition and reverses chronic renal injury. *Nat Med* 9, 964-968 (2003).

5. Danahay, H. *et al.* Notch2 is required for inflammatory cytokine-driven goblet cell metaplasia in the lung. *Cell Rep* 10, 239-252 (2015).

6. Quante, M. *et al.* Bile acid and inflammation activate gastric cardia stem cells in a mouse model of Barrett-like metaplasia. *Cancer Cell* 21, 36-51 (2012).

7. Menke, V. *et al.* Conversion of metaplastic Barrett's epithelium into post-mitotic goblet cells by gamma-secretase inhibition. *Disease models & mechanisms* 3, 104-110 (2010).

8. Terao, M. *et al.* Enhanced epithelial-mesenchymal transition-like phenotype in N-acetylglucosaminyltransferase V transgenic mouse skin promotes wound healing. *J Biol Chem* 286, 28303-28311 (2011).

9. Lv, Z.D. *et al.* Silencing of Prrx2 Inhibits the Invasion and Metastasis of Breast Cancer both In Vitro and In Vivo by Reversing Epithelial-Mesenchymal Transition. *Cell Physiol Biochem* 42, 1847-1856 (2017).

10. Wu, Y.Y. *et al.* SCUBE3 is an endogenous TGF-beta receptor ligand and regulates the epithelial-mesenchymal transition in lung cancer. *Oncogene* 30, 3682-3693 (2011).

11. Bonné, S. *et al.* Defining desmosomal plakophilin-3 interactions. *The Journal of Cell Biology* 161, 403-416 (2003).

12. Todorovic´, V., Koetsier, J.L., Godsel, L.M. & Green, K.J. Plakophilin 3 mediates Rap1-dependent desmosome assembly and adherens junction maturation. *Molecular Biology of the Cell* 25, 3749-3764 (2014).

13. Harrison, O.J. *et al.* Structural basis of adhesive binding by desmocollins and desmogleins. *Proc Natl Acad Sci U S A* 113, 7160-7165 (2016).

14. Wang, Y.W., Cheng, H.L., Ding, Y.R., Chou, L.H. & Chow, N.H. EMP1, EMP 2, and EMP3 as novel therapeutic targets in human cancer. *Biochim Biophys Acta* 1868, 199-211 (2017).

15. Boczonadi, V. & Maatta, A. Annexin A9 is a periplakin interacting partner in membrane-targeted cytoskeletal linker protein complexes. *FEBS Lett* 586, 3090-3096 (2012).

16. Otsubo, T. *et al.* Aberrant DNA hypermethylation reduces the expression of the desmosome-related molecule periplakin in esophageal squamous cell carcinoma. *Cancer Med* 4, 415-425 (2015).

17. Heller, E. & Fuchs, E. Tissue patterning and cellular mechanics. *J Cell Biol* 211, 219-231 (2015).

18. Flores-Tellez, T.N., Lopez, T.V., Vasquez Garzon, V.R. & Villa-Trevino, S. Co-Expression of Ezrin-CLIC5-Podocalyxin Is Associated with Migration and Invasiveness in Hepatocellular Carcinoma. *PLoS One* 10, e0131605 (2015).

19. Viswanatha, R., Bretscher, A. & Garbett, D. Dynamics of ezrin and EBP50 in regulating microvilli on the apical aspect of epithelial cells. *Biochem Soc Trans* 42, 189-194 (2014).

20. Cao, H.H. *et al.* A three-protein signature and clinical outcome in esophageal squamous cell carcinoma. *Oncotarget* 6, 5435-5448 (2015).

21. Tanaka, H. *et al.* Adherens junctions associated protein 1 serves as a predictor of recurrence of squamous cell carcinoma of the esophagus. *Int J Oncol* 47, 1811-1818 (2015).

22. Shen, Z.Y. *et al.* Upregulated expression of Ezrin and invasive phenotype in malignantly transformed esophageal epithelial cells. *World J Gastroenterol* 9, 1182-1186 (2003).

23. Schwebach, C.L., Agrawal, R., Lindert, S., Kudryashova, E. & Kudryashov, D.S. The Roles of Actin-Binding Domains 1 and 2 in the Calcium-Dependent Regulation of Actin Filament Bundling by Human Plastins. *J Mol Biol* 429, 2490-2508 (2017).

24. Hisano, T. *et al.* Increased expression of T-plastin gene in cisplatin-resistant human cancer cells: identification by mRNA differential display. *FEBS Lett* 397, 101-107 (1996).

25. Higuchi, Y. *et al.* Search for genes involved in UV-resistance in human cells by mRNA differential display: increased transcriptional expression of nucleophosmin and T-plastin genes in association with the resistance. *Biochem Biophys Res Commun* 248, 597-602 (1998).

26. Duggan, S.P. *et al.* The characterization of an intestine-like genomic signature maintained during Barrett's-associated adenocarcinogenesis reveals an NR5A2-mediated promotion of cancer cell survival. *Sci Rep* 6, 32638 (2016).

27. Khurana, S. & George, S.P. Regulation of cell structure and function by actin-binding proteins: villin's perspective. *FEBS Lett* 582, 2128-2139 (2008).

28. Liu, M. *et al.* RHOA GTPase Controls YAP-Mediated EREG Signaling in Small Intestinal Stem Cell Maintenance. *Stem Cell Reports* 9, 1961-1975 (2017).

29. Bruurs, L.J. *et al.* ATP8B1-mediated spatial organization of Cdc42 signaling maintains singularity during enterocyte polarization. *J Cell Biol* 210, 1055-1063 (2015).

30. Verhulst, P.M. *et al.* A flippase-independent function of ATP8B1, the protein affected in familial intrahepatic cholestasis type 1, is required for apical protein expression and microvillus formation in polarized epithelial cells. *Hepatology* 51, 2049-2060 (2010).

31. Croce, A. *et al.* A novel actin barbed-end-capping activity in EPS-8 regulates apical morphogenesis in intestinal cells of Caenorhabditis elegans. *Nat Cell Biol* 6, 1173-1179 (2004).

32. Tocchetti, A. *et al.* Loss of the actin remodeler Eps8 causes intestinal defects and improved metabolic status in mice. *PLoS One* 5, e9468 (2010).

33. Muller, T. *et al.* MYO5B mutations cause microvillus inclusion disease and disrupt epithelial cell polarity. *Nat Genet* 40, 1163-1165 (2008).

34. Giroux, V. *et al.* Long-lived keratin 15+ esophageal progenitor cells contribute to homeostasis and regeneration. *J Clin Invest* 127, 2378-2391 (2017).

35. Shearer, C., Going, J., Neilson, L., Mackay, C. & Stuart, R.C. Cytokeratin 7 and 20 expression in intestinal metaplasia of the distal oesophagus: relationship to gastro-oesophageal reflux disease. *Histopathology* 47, 268-275 (2005).

36. Nanashima, N., Horie, K., Yamada, T., Shimizu, T. & Tsuchida, S. Hair keratin KRT81 is expressed in normal and breast cancer cells and contributes to their invasiveness. *Oncol Rep* 37, 2964-2970 (2017).

37. Muckenhuber, A. *et al.* Pancreatic Ductal Adenocarcinoma Subtyping Using the Biomarkers Hepatocyte Nuclear Factor-1A and Cytokeratin-81 Correlates with Outcome and Treatment Response. *Clin Cancer Res* 24, 351-359 (2018).

38. Liu, S. *et al.* Identification of differentially expressed genes, lncRNAs and miRNAs which are associated with tumor malignant phenotypes in hepatoblastoma patients. *Oncotarget* 8, 97554-97564 (2017).

39. Rubinstein, E. *et al.* CD9, CD63, CD81, and CD82 are components of a surface tetraspan network connected to HLA-DR and VLA integrins. *Eur J Immunol* 26, 2657-2665 (1996).

40. Singethan, K. *et al.* CD9 clustering and formation of microvilli zippers between contacting cells regulates virus-induced cell fusion. *Traffic* 9, 924-935 (2008).

41. Zhu, J. *et al.* Prognostic role of CD82/KAI1 in multiple human malignant neoplasms: a meta-analysis of 31 studies. *Onco Targets Ther* 10, 5805-5816 (2017).

42. Sala-Valdes, M. *et al.* EWI-2 and EWI-F link the tetraspanin web to the actin cytoskeleton through their direct association with ezrin-radixin-moesin proteins. *J Biol Chem* 281, 19665-19675 (2006).

43. Pinto, D., Gregorieff, A., Begthel, H. & Clevers, H. Canonical Wnt signals are essential for homeostasis of the intestinal epithelium. *Genes Dev* 17, 1709-1713 (2003).

44. Almohazey, D. *et al.* The ErbB3 receptor tyrosine kinase negatively regulates Paneth cells by PI3K-dependent suppression of Atoh1. *Cell Death Differ* 24, 855-865 (2017).

45. Tanaka, M. *et al.* Spatial distribution and histogenesis of colorectal Paneth cell metaplasia in idiopathic inflammatory bowel disease. *J Gastroenterol Hepatol* 16, 1353-1359 (2001).

46. Miralles, F., Czernichow, P., Ozaki, K., Itoh, N. & Scharfmann, R. Signaling through fibroblast growth factor receptor 2b plays a key role in the development of the exocrine pancreas. *Proceedings of the National Academy of Sciences of the United States of America* 96, 6267-6272 (1999).

47. Yeh, J.C., Ong, E. & Fukuda, M. Molecular cloning and expression of a novel beta-1, 6-N-acetylglucosaminyltransferase that forms core 2, core 4, and I branches. *J Biol Chem* 274, 3215-3221 (1999).

48. Mukherjee, A. *et al.* Steroid receptor coactivator 2 is critical for progesterone-dependent uterine function and mammary morphogenesis in the mouse. *Mol Cell Biol* 26, 6571-6583 (2006).

49. Charytoniuk, D. *et al.* Intrastriatal sonic hedgehog injection increases Patched transcript levels in the adult rat subventricular zone. *Eur J Neurosci* 16, 2351-2357 (2002).

50. Wang, D.H. *et al.* Aberrant epithelial-mesenchymal Hedgehog signaling characterizes Barrett's metaplasia. *Gastroenterology* 138, 1810-1822 (2010).

51. Ramalho-Santos, M., Melton, D.A. & McMahon, A.P. Hedgehog signals regulate multiple aspects of gastrointestinal development. *Development* 127, 2763-2772 (2000).

52. Flanagan, D.J. *et al.* Frizzled7 functions as a Wnt receptor in intestinal epithelial Lgr5(+) stem cells. *Stem Cell Reports* 4, 759-767 (2015).

53. Farin, H.F., Van Es, J.H. & Clevers, H. Redundant sources of Wnt regulate intestinal stem cells and promote formation of Paneth cells. *Gastroenterology* 143, 1518-1529 e1517 (2012).

54. Farin, H.F. *et al.* Visualization of a short-range Wnt gradient in the intestinal stem-cell niche. *Nature* 530, 340-343 (2016).

55. Bakker, E.R. *et al.* Induced Wnt5a expression perturbs embryonic outgrowth and intestinal elongation, but is well-tolerated in adult mice. *Dev Biol* 369, 91-100 (2012).

56. Miyoshi, H., Ajima, R., Luo, C.T., Yamaguchi, T.P. & Stappenbeck, T.S. Wnt5a potentiates TGF-beta signaling to promote colonic crypt regeneration after tissue injury. *Science* 338, 108-113 (2012).

57. Mehdawi, L.M., Prasad, C.P., Ehrnstrom, R., Andersson, T. & Sjolander, A. Non-canonical WNT5A signaling up-regulates the expression of the tumor suppressor 15-PGDH and induces differentiation of colon cancer cells. *Mol Oncol* 10, 1415-1429 (2016).

58. Long, A. *et al.* WNT10A promotes an invasive and self-renewing phenotype in esophageal squamous cell carcinoma. *Carcinogenesis* 36, 598-606 (2015).

59. Grimont, A. *et al.* SOX9 regulates ERBB signalling in pancreatic cancer development. *Gut* 64, 1790-1799 (2015).

60. Mori-Akiyama, Y. *et al.* SOX9 is required for the differentiation of paneth cells in the intestinal epithelium. *Gastroenterology* 133, 539-546 (2007).

61. Herfs, M., Hubert, P. & Delvenne, P. Epithelial metaplasia: adult stem cell reprogramming and (pre)neoplastic transformation mediated by inflammation? *Trends Mol Med* 15, 245-253 (2009).

62. Que, J., Choi, M., Ziel, J.W., Klingensmith, J. & Hogan, B.L. Morphogenesis of the trachea and esophagus: current players and new roles for noggin and Bmps. *Differentiation* 74, 422-437 (2006).

63. Zhang, X., Westerhoff, M. & Hart, J. Expression of SOX9 and CDX2 in nongoblet columnar-lined esophagus predicts the detection of Barrett's esophagus during follow-up. *Mod Pathol* 28, 654-661 (2015).

64. Minacapelli, C.D. *et al.* Barrett's metaplasia develops from cellular reprograming of esophageal squamous epithelium due to gastroesophageal reflux. *Am J Physiol Gastrointest Liver Physiol* 312, G615-G622 (2017).

65. Clemons, N.J. *et al.* Sox9 drives columnar differentiation of esophageal squamous epithelium: a possible role in the pathogenesis of Barrett's esophagus. *Am J Physiol Gastrointest Liver Physiol* 303, G1335-1346 (2012).

66. Sock, E. *et al.* Gene targeting reveals a widespread role for the high-mobility-group transcription factor Sox11 in tissue remodeling. *Mol Cell Biol* 24, 6635-6644 (2004).

67. Lefebvre, V. The SoxD transcription factors--Sox5, Sox6, and Sox13--are key cell fate modulators. *Int J Biochem Cell Biol* 42, 429-432 (2010).

68. Li, J. *et al.* The clinical significance of circulating GPC1 positive exosomes and its regulative miRNAs in colon cancer patients. *Oncotarget* 8, 101189-101202 (2017).

69. Tanaka, M. *et al.* EVI1 modulates oncogenic role of GPC1 in pancreatic carcinogenesis. *Oncotarget* 8, 99552-99566 (2017).

70. Hara, H. *et al.* Overexpression of glypican-1 implicates poor prognosis and their chemoresistance in oesophageal squamous cell carcinoma. *Br J Cancer* 115, 66-75 (2016).

71. Bosse, K.R. *et al.* Identification of GPC2 as an Oncoprotein and Candidate Immunotherapeutic Target in High-Risk Neuroblastoma. *Cancer Cell* 32, 295-309 e212 (2017).

72. Filmus, J., Church, J.G. & Buick, R.N. Isolation of a cDNA corresponding to a developmentally regulated transcript in rat intestine. *Mol Cell Biol* 8, 4243-4249 (1988).

73. Farkas, S.A., Vymetalkova, V., Vodickova, L., Vodicka, P. & Nilsson, T.K. DNA methylation changes in genes frequently mutated in sporadic colorectal cancer and in the DNA repair and Wnt/beta-catenin signaling pathway genes. *Epigenomics* 6, 179-191 (2014).

74. Dai, Y. *et al.* Genome-Wide Analysis of Barrett's Adenocarcinoma. A First Step Towards Identifying Patients at Risk and Developing Therapeutic Paths. *Transl Oncol* 11, 116-124 (2017).

75. Li, Y. *et al.* Immune signature profiling identified predictive and prognostic factors for esophageal squamous cell carcinoma. *Oncoimmunology* 6, e1356147 (2017).

76. Yoon, N.Y. *et al.* Simultaneous detection of barrier- and immune-related gene variations in patients with atopic dermatitis by reverse blot hybridization assay. *Clin Exp Dermatol* (2018).

77. Pham, T.A. *et al.* Epithelial IL-22RA1-mediated fucosylation promotes intestinal colonization resistance to an opportunistic pathogen. *Cell Host Microbe* 16, 504-516 (2014).

78. Morrison, P.J. *et al.* Differential Requirements for IL-17A and IL-22 in Cecal versus Colonic Inflammation Induced by Helicobacter hepaticus. *Am J Pathol* 185, 3290-3303 (2015).

79. Yao, Q. *et al.* Synergistic role of Caspase-8 and Caspase-3 expressions: Prognostic and predictive biomarkers in colorectal cancer. *Cancer Biomark* (2018).

80. Shea-Donohue, T. *et al.* Mice deficient in the CXCR2 ligand, CXCL1 (KC/GRO-alpha), exhibit increased susceptibility to dextran sodium sulfate (DSS)-induced colitis. *Innate Immun* 14, 117-124 (2008).

81. Lin, Y., Devin, A., Rodriguez, Y. & Liu, Z.G. Cleavage of the death domain kinase RIP by caspase-8 prompts TNF-induced apoptosis. *Genes Dev* 13, 2514-2526 (1999).

82. Feng, S. *et al.* Cleavage of RIP3 inactivates its caspase-independent apoptosis pathway by removal of kinase domain. *Cell Signal* 19, 2056-2067 (2007).

83. Seifert, L. *et al.* The necrosome promotes pancreatic oncogenesis via CXCL1 and Mincle-induced immune suppression. *Nature* 532, 245-249 (2016).

84. Moriwaki, K., Balaji, S. & Chan, F.K. Border Security: The Role of RIPK3 in Epithelium Homeostasis. *Front Cell Dev Biol* 4, 70 (2016).

85. Han, G. *et al.* Effect of Annexin A1 gene on the proliferation and invasion of esophageal squamous cell carcinoma cells and its regulatory mechanisms. *Int J Mol Med* 39, 357-363 (2017).

86. Wang, K.L. *et al.* Expression of annexin A1 in esophageal and esophagogastric junction adenocarcinomas: association with poor outcome. *Clin Cancer Res* 12, 4598-4604 (2006).

87. Zhang, M. *et al.* Artemin is hypoxia responsive and promotes oncogenicity and increased tumor initiating capacity in hepatocellular carcinoma. *Oncotarget* 7, 3267-3282 (2016).

88. Paterson, A.L. *et al.* Characterization of the timing and prevalence of receptor tyrosine kinase expression changes in oesophageal carcinogenesis. *J Pathol* 230, 118-128 (2013).

89. Manfredi, J.J. Tumor suppression by p53 involves inhibiting an enabler, FGF13. *Proceedings of the National Academy of Sciences of the United States of America* 114, 632-633 (2017).

90. Yamashita, K., Upadhay, S., Mimori, K., Inoue, H. & Mori, M. Clinical significance of secreted protein acidic and rich in cystein in esophageal carcinoma and its relation to carcinoma progression. *Cancer* 97, 2412-2419 (2003).

91. Feng, M. *et al.* Store-independent activation of Orai1 by SPCA2 in mammary tumors. *Cell* 143, 84-98 (2010).

92. Andoh, A., Kinoshita, K., Rosenberg, I. & Podolsky, D.K. Intestinal trefoil factor induces decay-accelerating factor expression and enhances the protective activities against complement activation in intestinal epithelial cells. *J Immunol* 167, 3887-3893 (2001).

93. Fang, Y. & Zhang, X. Targeting NEK2 as a promising therapeutic approach for cancer treatment. *Cell Cycle* 15, 895-907 (2016).

94. Hu, S. *et al.* miR-532 promoted gastric cancer migration and invasion by targeting NKD1. *Life Sci* 177, 15-19 (2017).

95. Wang, C. *et al.* Long non-coding RNA HNF1A-AS1 promotes hepatocellular carcinoma cell proliferation by repressing NKD1 and P21 expression. *Biomed Pharmacother* 89, 926-932 (2017).

96. Zhang, S., Li, J. & Wang, X. NKD1 correlates with a poor prognosis and inhibits cell proliferation by inducing p53 expression in hepatocellular carcinoma. *Tumour Biol* 37, 14059-14067 (2016).

97. Leung, C.O.-n. *et al.* PIM1 regulates glycolysis and promotes tumor progression in hepatocellular carcinoma. *Oncotarget* 6, 10880-10892 (2015).

98. Warfel, N.A. & Kraft, A.S. PIM kinase (and Akt) biology and signaling in tumors. *Pharmacology & therapeutics* 151, 41-49 (2015).

99. Younes, M. *et al.* Relationship between dysplasia, p53 protein accumulation, DNA ploidy, and Glut1 overexpression in Barrett metaplasia. *Scand J Gastroenterol* 35, 131-137 (2000).

100. Berlth, F. *et al.* Both GLUT-1 and GLUT-14 are Independent Prognostic Factors in Gastric Adenocarcinoma. *Ann Surg Oncol* 22 Suppl 3, S822-831 (2015).

101. Shen, Y.M., Arbman, G., Olsson, B. & Sun, X.F. Overexpression of GLUT1 in colorectal cancer is independently associated with poor prognosis. *Int J Biol Markers* 26, 166-172 (2011).

102. Zheng, L.S. *et al.* SPINK6 Promotes Metastasis of Nasopharyngeal Carcinoma via Binding and Activation of Epithelial Growth Factor Receptor. *Cancer Res* 77, 579-589 (2017).
