# Supplementary material for: HOXA13 in etiology and oncogenic potential of Barrett’s esophagus
Source: Nat Commun. 2021 Jun 7;12:3354. doi: 10.1038/s41467-021-23641-8 (PMC8184780; doi:10.1038/s41467-021-23641-8)
Supplement: Supplementary file 6 — Supplementary Dataset 3 [file 41467_2021_23641_MOESM6_ESM.zip › 193347_3_data_set_5347017_qhxyhz.docx]

| Gene | Fold Change and q of *HOXA13*^+^ vs *HOXA13*^-^ BAR-T cells | | Known function | Detailed description |
| --- | --- | --- | --- | --- |
| All multiple testing corrected, significantly regulated genes, in the same direction in both the BAR-T and EPC2-hTERT datasets. | | | | |
| *IL7R* | 3.07 | 0.00 | immunity | Interleukin 7 receptor, blockade of the IL-7/IL-7R signaling pathway was found to render T cell-deficient mice more sensitive to chemically-induced intestinal epithelial cell damage and subsequent colitis ^1^. |
| *FAM196B* | 2.32 | 0.02 | cancer | Family with sequence similarity 196 member B, was found to promote proliferation of gastric cancer cells through the AKT signaling pathway ^2^. |
| *ADAMTS6* | 2.13 | 0.03 | cancer | ADAM metallopeptidase with thrombospondin type 1 motif 6, is upregulated in breast cancer where it was found to suppresses tumor progression via the ERK signaling pathway ^3, 4^. |
| *NRG1* | 2.07 | 0.02 | cancer | Neuregulin 1, ligand of ERBB3, paracrine NRG1/ERBB3 signaling was found to promote CRC cell progression, and high NRG1 expression is associated with poor prognosis in CRC ^5^. |
| *LTBP1* | 1.94 | 0.02 | cancer | Latent transforming growth factor β binding protein 1, its expression increases with glioma progression ^6^. |
| *JAG1* | 1.85 | 0.04 | morphology | Jagged 1, is a ligand of the NOTCH pathway, this pathway was found to be associated with malignant transformation ^7-9^. |
| *ELL2* | 1.82 | 0.02 | cancer | Elongation factor for RNA polymerase 2, was found to be part of the super elongation complex and in combination with *HOXA9* and *10* associated with leukemia. ELL2 regulates DNA non-homologous end joining repair in prostate cancer cells ^10^. |
| *SMAD7* | 1.80 | 0.05 | cancer/immunity | SMAD family member 7, is a TGFβ type 1 receptor antagonist. It was found to block TGFβ1 and activing from binding to the receptor, which prevents access to SMAD2 ^11^. |
| *C12orf75* | 1.78 | 0.03 | cancer | Chromosome 12 open reading frame 75, is overexpressed in CRC, and upregulates the Wnt pathway ^12^. |
| *AXL* | 1.70 | 0.04 | immune regulation, cancer | AXL receptor tyrosine kinase, was found to be a negative predictor of survival in EAC and ESCC ^13^. AXL also contributes to a reduction in inflammation (Dransfield and Farnworth, 2016). |
| *TIPARP* | 1.57 | 0.00 | cancer | TCDD inducible poly(ADP-ribose) polymerase, was found to be overexpressed in meningioma and its locus was associated with ovarian cancer in a GWAS ^14, 15^. |
| *IKBIP* | 1.56 | 0.02 | cancer | Inhibitor of nuclear factor kappa-B kinase-interacting protein, was found to be a TP53 target gene with pro-apoptotic function ^16^. |
| *DUSP7* | 1.52 | 0.04 | cancer | Dual specificity phosphatase 7, was found to be overexpressed in myeloid leukemia and other malignancies ^17^. |
| *GOLIM4* | 1.47 | 0.04 | morphology | Golgi integral membrane protein 4, was found to be essential for podycyte cytoskeleton ^18^. It mediates endosome exit ^19^. |
| *FUCA1* | 0.69 | 0.02 | cancer | α-L-fucosidase 1, loss-of-function mutations are found in several cancers, its expression is reduced in CRC, and low expression is associated with poorer prognosis in several cancers ^20^. Has high expression in the distal vs the proximal GI-tract ^21^. |
| *MAP3K5* | 0.63 | 0.04 | immune regulation, cancer | Mitogen-activated protein kinase kinase kinase 5, also called apoptosis signal-regulating kinase 1. Knockdown of *MAP3K5* blocked dextran sulfate sodium induced tight junction disruption and subsequent barrier dysfunction ^22^. |
| *EXPH5* | 0.56 | 0.04 | morphology | Exophilin 5, mutations result in a skin fragility phenotype ^23^. |
| *C6orf132* | 0.56 | 0.03 | unknown | Chromosome 6 open reading frame 132. |
| *HCAR2* | 0.50 | 0.05 | cancer/immunity | Hydroxycarboxylic acid receptor 2, is a G-protein-coupled receptor for the bacterial fermentation product butyrate and was found to function as a tumor suppressor in colon ^24, 25^. |
| *RAB27B* | 0.50 | 0.03 | cancer | RAB27B, member RAS oncogene family, decreased expression was found to correlate with metastasis and poor prognosis in CRC ^26^. However, it prevented cancer invasion and proliferation in pancreatic ductal AC cells ^27^. |
| *TNFAIP2* | 0.49 | 0.03 | cancer | TNFα induced protein 2, downregulation was found to suppress proliferation and metastasis in ESCC through activation of the Wnt/β-catenin signaling pathway ^28^. |
| *MATN2* | 0.49 | 0.04 | cancer | Matrilin 2, was identified as a tumor suppressor in HCC ^29^. |
| *TTC9* | 0.48 | 0.02 | cancer | Tetratricopeptide repeat domain 9, up-regulation is coupled with progesterone-mediated growth inhibition and induction of focal adhesion ^30^. |
| *TMPRSS4* | 0.48 | 0.02 | cancer | Transmembrane serine protease 4, overexpression appears a poor prognostic factor for solid tumors ^31^. |
| *ANXA9* | 0.48 | 0.04 | morphology | Annexin A9, and periplakin (*PPL*; FC0.57, *q*0.21, *p*0.01) co-localize in the epidermis, *ANXA9* was found upregulated in differentiating keratinocytes ^32^. |
| *KLK7* | 0.42 | 0.01 | morphology | Kallikrein related peptidase 7, also called stratum corneum chymotryptic enzyme is a proteinase generally present in the stratum corneum ^33^. |
| *MYO5C* | 0.42 | 0.01 | morphology | Myosin VC, is involved in actin mediated membrane trafficking. *Myo5c* appears to mediate apical exocytosis of secretory vesicles ^34, 35^. |
| *SERPINB13* | 0.38 | 0.00 | cancer | Serpin family B member 13, was found downregulated in many types of cancer, its expression in head and neck SCC associates with poor clinical outcome (de Koning et al., 2009; Shiiba et al., 2010). |
| Morphology associated genes from the BAR-T dataset. | | | | |
| *MCAM* | 3.47 | 0.00 | morphology | Melanoma cell adhesion molecule, it mediates the extension of microvilli in mouse melanoma cell lines ^36^. When subjects with fatal asthma are compared to controls it is located at the brush border of airway epithelium of the subjects ^37^. |
| *SHROOM4* | 2.54 | 0.00 | morphology | Shroom family member 4, was found to be regulator of cyto-skeletal architecture ^38^. In Xenopus, cells expressing Shroom family of protein members tend to be elongated, this is associated with microtubule bundles arranged along the apico-basal axis ^39^. |
| *NIPAL4* | 2.05 | 0.00 | morphology | NIPA like domain containing 4, also called Ichthyin, is mutated in ichthyosis. In *Nipal4* knock-out mice, the number of stratum corneum layers was from 10 to 20, indicative of hyperkeratosis ^40^. |
| *TMOD2* | 1.74 | 0.02 | morphology | Tropomodulin 2, is a neuronal-specific actin-regulatory protein, was found to cap the pointed end of actin filaments preventing both elongation and depolymerization ^41^. |
| *EXPH5* | 0.56 | 0.04 | morphology | Exophilin 5, see above. |
| *FBLN1* | 0.52 | 0.02 | morphology | Fibulin-1, is a secreted extracellular matrix glycoprotein ^42, 43^. |
| *MPP7* | 0.51 | 0.02 | morphology | Membrane palmitoylated protein 7, forms a complex with the polarity protein *DLG1* and was found to facilitate epithelial cell polarity and tight junction formation ^44^. It is overexpressed in metaplasia ^45, 46^. |
| *SPTBN2* | 0.47 | 0.02 | morphology | Spectrin β, non-erythrocytic 2, is a components of a cell's membrane-cytoskeleton ^47^. |
| *MUC5AC* | 0.37 | 0.00 | morphology | Mucin 5AC, oligomeric mucus/gel-forming, is a gastric mucin ^21^. |
| *ITGB7* | 0.34 | 0.00 | morphology | Integrin subunit β 7, affects multiple myeloma-cell adhesion and migration ^48^. |
| Cancer associated genes from the BAR-T dataset. | | | | |
| *DLL1* | 2.57 | 0.00 | pro-oncogenic | Notch signaling was found to be associated with malignant transformation at the GEJ ^8, 49^ |
| *MAF* | 2.39 | 0.00 | pro-oncogenic | MAF bZIP transcription factor, also called c-MAF, was found to be a mediator of bone metastasis in breast cancer ^50^. Overexpressing c-Maf enhanced cholangiocarcinoma growth in mice ^51^. |
| *FAM196B* | 2.32 | 0.02 | pro-oncogenic | Family with sequence similarity 196 member B, see above. |
| *EPGN* | 2.23 | 0.03 | pro-oncogenic | Epithelial mitogen, was found to be an activator of Erbb1 and can act as a mitogen ^52^. |
| *FBXO2* | 2.19 | 0.02 | pro-oncogenic | F-box protein 2, the overexpression of which was found to induce EMT in gastric cancer cells, and is associated with more lymph node metastasis and shorter overall survival ^53^. |
| *ADAMTS6* | 2.13 | 0.03 | pro-oncogenic | ADAM metallopeptidase with thrombospondin type 1 motif 6, see above. |
| *BCL11B* | 2.12 | 0.01 | pro-oncogenic | B cell CLL/lymphoma 11B, its impairment was found to promote tumor development in mouse and human intestine ^54^. On the other hand, it was identified as a potential oncogene in AML ^55^. |
| *NRG1* | 2.07 | 0.02 | pro-oncogenic | Neuregulin 1, see above. |
| *NDRG1* | 2.05 | 0.00 | anti-oncogenic | N-myc downstream regulated 1, was found to be a potent, iron-regulated growth and metastasis suppressor that was found to be negatively correlated with cancer progression in a number of tumors ^56^. |
| *TGFBI* | 2.03 | 0.02 | pro-oncogenic | Transforming growth factor β induced, was found to promote the growth of GI-tract tumors, specifically ESCC and gastric cancer ^57^. |
| *IL1B* | 2.00 | 0.00 | pro-oncogenic | Interleukin 1 β, was found to be sufficient to induce esophagitis, BE, and EAC in a mouse model (Quante et al., 2012). *MMP9* (FC2.06, p=0.001, q=0.051) could upregulate Il1β in the mouse (Fang et al., 2017). |
| *LAMC2* | 1.94 | 0.00 | pro-oncogenic | Laminin subunit γ 2, in ESCC and CRC *LAMC2* is upregulated and was found to be associated with worse survival ^58, 59^. |
| *CD274* | 1.90 | 0.03 | pro-oncogenic | CD274 molecule, also called programmed cell death 1 ligand 1, and programmed cell death 1 ligand 2 (*PDCD1LG2*; FC=1.99, *q*=0.053, *p*=0.00) were both upregulated. |
| *GRB10* | 1.87 | 0.00 | pro-oncogenic | Growth factor receptor bound protein 10, is upregulated in CRC vs peritumor tissue ^60^. Overexpression in CRC was found to be associated with decreased survival ^61^. |
| *ELL2* | 1.82 | 0.02 | pro-oncogenic | Elongation factor for RNA polymerase II 2, see above. |
| *SMAD7* | 1.80 | 0.05 | unknown | SMAD family member 7, see above |
| *C12orf75* | 1.78 | 0.03 | pro-oncogenic | Chromosome 12 open reading frame 75, see above. |
| *ITGA5* | 1.77 | 0.00 | pro-oncogenic | Integrin subunit α 5, overexpression of the ITGA5 is correlated with an increased risk of perineural invasion in CRC ^62^. ITGA5 was found to modulate apoptosis, adhesion, migration, and facilitates cancer cell invasion through enhanced contractile forces ^63, 64^. |
| *AXL* | 1.70 | 0.04 | pro-oncogenic | AXL receptor tyrosine kinase, see above. |
| *DUSP7* | 1.52 | 0.04 | pro-oncogenic | Dual specificity phosphatase 7, see above. |
| *LPCAT1* | 1.50 | 0.02 | pro-oncogenic | Lysophosphatidylcholine acyltransferase 1, its expression was higher in CRC vs normal mucosa. A CRC cell line with overexpression, SW480, were found to significantly increase their growth rate ^65^. |
| *FURIN* | 1.49 | 0.03 | pro-oncogenic | Notch signaling was found to be associated with malignant transformation at the GEJ ^8, 49^. |
| *MAP3K5* | 0.63 | 0.04 | unknown | Mitogen-activated protein kinase kinase kinase 5, see above. |
| *CTSD* | 0.53 | 0.03 | unknown | Cathepsin D, can either induce apoptosis in presence of cytotoxic factors, but in certain studies an inhibitory role in apoptosis was also reviewed ^66^. |
| *APOBEC3B* | 0.50 | 0.02 | anti-oncogenic | Apolipoprotein B mRNA editing enzyme catalytic subunit 3B, was found to be an enzymatic source of mutations in various cancer types ^67^. |
| *HCAR2* | 0.50 | 0.05 | pro-oncogenic | Hydroxycarboxylic acid receptor 2, see above. |
| *RAB27B* | 0.50 | 0.03 | anti-oncogenic | RAB27B, member RAS oncogene family, see above. |
| *PHLPP1* | 0.49 | 0.02 | pro-oncogenic | PH domain and leucine rich repeat protein phosphatase 1, is an important regulator of Akt serine-threonine kinases and protein kinase C isoforms. It may act as a tumor suppressor in several types of cancer due to its ability to block growth factor-induced signaling in cancer cells ^68, 69^. |
| *TNFAIP2* | 0.49 | 0.03 | anti-oncogenic | TNFα induced protein 2, see above. |
| *TMPRSS4* | 0.48 | 0.02 | anti-oncogenic | Transmembrane serine protease 4, see above. |
| *RTKN2* | 0.47 | 0.02 | anti-oncogenic | Rhotekin 2, was found to be increase proliferation and reduces apoptosis in pancreatic cancer, CRC, and HCC ^70-72^. |
| *LTBP3* | 0.37 | 0.00 | pro-oncogenic | Latent-transforming growth factor β-binding protein 3, promotes early metastatic events ^73, 74^. |

Multiple testing corrected significantly differentially regulated genes in the same direction in both the BAR-T and EPC2-hTERT dataset (n=28); molecules which have been associated with morphological characteristics in literature; molecules, not described in the main text, which are associated in literature with oncogenic characteristics are described in more detail. All FC and *q* values shown pertain to the BAR-T dataset. Information in the “known function” and “Detailed description” columns was obtained through non-systematic review and should not be considered as an exhaustive overview of the literature.

Reference list:

1. Shalapour, S. *et al.* Interleukin-7 links T lymphocyte and intestinal epithelial cell homeostasis. *PLoS One* 7, e31939 (2012).

2. Zhang, J. *et al.* FAM196B acts as oncogene and promotes proliferation of gastric cancer cells through AKT signaling pathway. *Cell Mol Biol (Noisy-le-grand)* 63, 18-23 (2017).

3. Porter, S. *et al.* Dysregulated expression of adamalysin-thrombospondin genes in human breast carcinoma. *Clin Cancer Res* 10, 2429-2440 (2004).

4. Xie, Y. *et al.* ADAMTS6 suppresses tumor progression via the ERK signaling pathway and serves as a prognostic marker in human breast cancer. *Oncotarget* 7, 61273-61283 (2016).

5. De Boeck, A. *et al.* Bone marrow-derived mesenchymal stem cells promote colorectal cancer progression through paracrine neuregulin 1/HER3 signalling. *Gut* 62, 550-560 (2013).

6. Tritschler, I. *et al.* Modulation of TGF-beta activity by latent TGF-beta-binding protein 1 in human malignant glioma cells. *Int J Cancer* 125, 530-540 (2009).

7. Danahay, H. *et al.* Notch2 is required for inflammatory cytokine-driven goblet cell metaplasia in the lung. *Cell Rep* 10, 239-252 (2015).

8. Quante, M. *et al.* Bile acid and inflammation activate gastric cardia stem cells in a mouse model of Barrett-like metaplasia. *Cancer Cell* 21, 36-51 (2012).

9. Menke, V. *et al.* Conversion of metaplastic Barrett's epithelium into post-mitotic goblet cells by gamma-secretase inhibition. *Disease models & mechanisms* 3, 104-110 (2010).

10. Zang, Y. *et al.* ELL2 regulates DNA non-homologous end joining (NHEJ) repair in prostate cancer cells. *Cancer Lett* 415, 198-207 (2018).

11. Liu, X. *et al.* Smad7 but not Smad6 cooperates with oncogenic ras to cause malignant conversion in a mouse model for squamous cell carcinoma. *Cancer Res* 63, 7760-7768 (2003).

12. Najafi, H., Soltani, B.M., Dokanehiifard, S., Nasiri, S. & Mowla, S.J. Alternative splicing of the OCC-1 gene generates three splice variants and a novel exonic microRNA, which regulate the Wnt signaling pathway. *Rna* 23, 70-85 (2017).

13. Zhang, S. *et al.* The prognostic role of Gas6/Axl axis in solid malignancies: a meta-analysis and literature review. *Onco Targets Ther* 11, 509-519 (2018).

14. Talari, N.K., Panigrahi, M.K., Madigubba, S. & Phanithi, P.B. Overexpression of aryl hydrocarbon receptor (AHR) signalling pathway in human meningioma. *J Neurooncol* (2018).

15. Goode, E.L. *et al.* A genome-wide association study identifies susceptibility loci for ovarian cancer at 2q31 and 8q24. *Nat Genet* 42, 874-879 (2010).

16. Hofer-Warbinek, R. *et al.* A highly conserved proapoptotic gene, IKIP, located next to the APAF1 gene locus, is regulated by p53. *Cell Death Differ* 11, 1317-1325 (2004).

17. Lountos, G.T., Austin, B.P., Tropea, J.E. & Waugh, D.S. Structure of human dual-specificity phosphatase 7, a potential cancer drug target. *Acta Crystallogr F Struct Biol Commun* 71, 650-656 (2015).

18. Lu, Y. *et al.* Genome-wide identification of genes essential for podocyte cytoskeletons based on single-cell RNA sequencing. *Kidney Int* 92, 1119-1129 (2017).

19. Natarajan, R. & Linstedt, A.D. A cycling cis-Golgi protein mediates endosome-to-Golgi traffic. *Mol Biol Cell* 15, 4798-4806 (2004).

20. Ezawa, I. *et al.* Novel p53 target gene FUCA1 encodes a fucosidase and regulates growth and survival of cancer cells. *Cancer Sci* 107, 734-745 (2016).

21. Uhlen, M. *et al.* Proteomics. Tissue-based map of the human proteome. *Science* 347, 1260419 (2015).

22. Samak, G. *et al.* Calcium/Ask1/MKK7/JNK2/c-Src signalling cascade mediates disruption of intestinal epithelial tight junctions by dextran sulfate sodium. *Biochem J* 465, 503-515 (2015).

23. Malchin, N. *et al.* A novel homozygous deletion in EXPH5 causes a skin fragility phenotype. *Clin Exp Dermatol* 41, 915-918 (2016).

24. Thangaraju, M. *et al.* GPR109A is a G-protein-coupled receptor for the bacterial fermentation product butyrate and functions as a tumor suppressor in colon. *Cancer Res* 69, 2826-2832 (2009).

25. Singh, N. *et al.* Activation of Gpr109a, receptor for niacin and the commensal metabolite butyrate, suppresses colonic inflammation and carcinogenesis. *Immunity* 40, 128-139 (2014).

26. Dong, W. *et al.* Decreased expression of Rab27A and Rab27B correlates with metastasis and poor prognosis in colorectal cancer. *Discov Med* 20, 357-367 (2015).

27. Li, J., Jin, Q., Huang, F., Tang, Z. & Huang, J. Effects of Rab27A and Rab27B on Invasion, Proliferation, Apoptosis, and Chemoresistance in Human Pancreatic Cancer Cells. *Pancreas* 46, 1173-1179 (2017).

28. Xie, Y. & Wang, B. Downregulation of TNFAIP2 suppresses proliferation and metastasis in esophageal squamous cell carcinoma through activation of the Wnt/beta-catenin signaling pathway. *Oncol Rep* 37, 2920-2928 (2017).

29. Fullar, A. *et al.* Lack of Matrilin-2 favors liver tumor development via Erk1/2 and GSK-3beta pathways in vivo. *PLoS One* 9, e93469 (2014).

30. Cao, S., Iyer, J.K. & Lin, V. Identification of tetratricopeptide repeat domain 9, a hormonally regulated protein. *Biochem Biophys Res Commun* 345, 310-317 (2006).

31. Zeng, P. *et al.* TMPRSS4 as an emerging potential poor prognostic factor for solid tumors: A systematic review and meta-analysis. *Oncotarget* 7, 76327-76336 (2016).

32. Boczonadi, V. & Maatta, A. Annexin A9 is a periplakin interacting partner in membrane-targeted cytoskeletal linker protein complexes. *FEBS Lett* 586, 3090-3096 (2012).

33. Lundstrom, A. & Egelrud, T. Stratum corneum chymotryptic enzyme: a proteinase which may be generally present in the stratum corneum and with a possible involvement in desquamation. *Acta Derm Venereol* 71, 471-474 (1991).

34. Marchelletta, R.R., Jacobs, D.T., Schechter, J.E., Cheney, R.E. & Hamm-Alvarez, S.F. The class V myosin motor, myosin 5c, localizes to mature secretory vesicles and facilitates exocytosis in lacrimal acini. *Am J Physiol Cell Physiol* 295, C13-28 (2008).

35. Sladewski, T.E., Krementsova, E.B. & Trybus, K.M. Myosin Vc Is Specialized for Transport on a Secretory Superhighway. *Curr Biol* 26, 2202-2207 (2016).

36. Okumura, S. *et al.* Involvement of gicerin in the extension of microvilli. *Exp Cell Res* 271, 269-276 (2001).

37. Simon, G.C. *et al.* Up-regulation of MUC18 in airway epithelial cells by IL-13: implications in bacterial adherence. *Am J Respir Cell Mol Biol* 44, 606-613 (2011).

38. Yoder, M. & Hildebrand, J.D. Shroom4 (Kiaa1202) is an actin-associated protein implicated in cytoskeletal organization. *Cell Motil Cytoskeleton* 64, 49-63 (2007).

39. Lee, C., Le, M.P. & Wallingford, J.B. The shroom family proteins play broad roles in the morphogenesis of thickened epithelial sheets. *Dev Dyn* 238, 1480-1491 (2009).

40. Honda, Y. *et al.* Decreased Skin Barrier Lipid Acylceramide and Differentiation-Dependent Gene Expression in Ichthyosis Gene Nipal4 Knockout Mice. *J Invest Dermatol* (2017).

41. Arslan, B., Colpan, M., Gray, K.T., Abu-Lail, N.I. & Kostyukova, A.S. Characterizing interaction forces between actin and proteins of the tropomodulin family reveals the presence of the N-terminal actin-binding site in leiomodin. *Arch Biochem Biophys* 638, 18-26 (2018).

42. Timpl, R., Sasaki, T., Kostka, G. & Chu, M.L. Fibulins: a versatile family of extracellular matrix proteins. *Nat Rev Mol Cell Biol* 4, 479-489 (2003).

43. Balbona, K. *et al.* Fibulin binds to itself and to the carboxyl-terminal heparin-binding region of fibronectin. *J Biol Chem* 267, 20120-20125 (1992).

44. Stucke, V.M., Timmerman, E., Vandekerckhove, J., Gevaert, K. & Hall, A. The MAGUK protein MPP7 binds to the polarity protein hDlg1 and facilitates epithelial tight junction formation. *Mol Biol Cell* 18, 1744-1755 (2007).

45. Botelho, N.K. *et al.* Gene expression alterations in formalin-fixed, paraffin-embedded Barrett esophagus and esophageal adenocarcinoma tissues. *Cancer Biol Ther* 10, 172-179 (2010).

46. Shimizu, T. *et al.* Characterization of progressive metaplasia in the gastric corpus mucosa of Mongolian gerbils infected with Helicobacter pylori. *J Pathol* 239, 399-410 (2016).

47. Naydenov, N.G. & Ivanov, A.I. Adducins regulate remodeling of apical junctions in human epithelial cells. *Mol Biol Cell* 21, 3506-3517 (2010).

48. Neri, P. *et al.* Integrin beta7-mediated regulation of multiple myeloma cell adhesion, migration, and invasion. *Blood* 117, 6202-6213 (2011).

49. Menke, V. *et al.* Conversion of metaplastic Barrett's epithelium into post-mitotic goblet cells by gamma-secretase inhibition. *Dis Model Mech* 3, 104-110 (2010).

50. Pavlovic, M. *et al.* Enhanced MAF Oncogene Expression and Breast Cancer Bone Metastasis. *J Natl Cancer Inst* 107, djv256 (2015).

51. Yang, H. *et al.* Deregulated methionine adenosyltransferase alpha1, c-Myc, and Maf proteins together promote cholangiocarcinoma growth in mice and humans(double dagger). *Hepatology* 64, 439-455 (2016).

52. Kochupurakkal, B.S. *et al.* Epigen, the last ligand of ErbB receptors, reveals intricate relationships between affinity and mitogenicity. *J Biol Chem* 280, 8503-8512 (2005).

53. Sun, X. *et al.* FBXO2, a novel marker for metastasis in human gastric cancer. *Biochem Biophys Res Commun* 495, 2158-2164 (2018).

54. Sakamaki, A. *et al.* Bcl11b SWI/SNF-complex subunit modulates intestinal adenoma and regeneration after gamma-irradiation through Wnt/beta-catenin pathway. *Carcinogenesis* 36, 622-631 (2015).

55. Abbas, S. *et al.* Integrated genome-wide genotyping and gene expression profiling reveals BCL11B as a putative oncogene in acute myeloid leukemia with 14q32 aberrations. *Haematologica* 99, 848-857 (2014).

56. Bae, D.H. *et al.* The role of NDRG1 in the pathology and potential treatment of human cancers. *J Clin Pathol* 66, 911-917 (2013).

57. Yokobori, T. & Nishiyama, M. TGF-beta Signaling in Gastrointestinal Cancers: Progress in Basic and Clinical Research. *J Clin Med* 6 (2017).

58. Huang, D., Du, C., Ji, D., Xi, J. & Gu, J. Overexpression of LAMC2 predicts poor prognosis in colorectal cancer patients and promotes cancer cell proliferation, migration, and invasion. *Tumour Biol* 39, 1010428317705849 (2017).

59. Shou, J.Z. *et al.* Overexpression of CDC25B and LAMC2 mRNA and protein in esophageal squamous cell carcinomas and premalignant lesions in subjects from a high-risk population in China. *Cancer Epidemiol Biomarkers Prev* 17, 1424-1435 (2008).

60. Zhang, T., Ma, Y., Fang, J., Liu, C. & Chen, L. A Deregulated PI3K-AKT Signaling Pathway in Patients with Colorectal Cancer. *J Gastrointest Cancer* (2017).

61. Qi, L. & Ding, Y. Screening and regulatory network analysis of survival-related genes of patients with colorectal cancer. *Sci China Life Sci* 57, 526-531 (2014).

62. Viana Lde, S. *et al.* Relationship between the expression of the extracellular matrix genes SPARC, SPP1, FN1, ITGA5 and ITGAV and clinicopathological parameters of tumor progression and colorectal cancer dissemination. *Oncology* 84, 81-91 (2013).

63. Mierke, C.T., Frey, B., Fellner, M., Herrmann, M. & Fabry, B. Integrin alpha5beta1 facilitates cancer cell invasion through enhanced contractile forces. *J Cell Sci* 124, 369-383 (2011).

64. Desgrosellier, J.S. & Cheresh, D.A. Integrins in cancer: biological implications and therapeutic opportunities. *Nat Rev Cancer* 10, 9-22 (2010).

65. Mansilla, F. *et al.* Lysophosphatidylcholine acyltransferase 1 (LPCAT1) overexpression in human colorectal cancer. *J Mol Med (Berl)* 87, 85-97 (2009).

66. Minarowska, A., Minarowski, L., Karwowska, A. & Gacko, M. Regulatory role of cathepsin D in apoptosis. *Folia Histochem Cytobiol* 45, 159-163 (2007).

67. Zou, J., Wang, C., Ma, X., Wang, E. & Peng, G. APOBEC3B, a molecular driver of mutagenesis in human cancers. *Cell Biosci* 7, 29 (2017).

68. Brognard, J. & Newton, A.C. PHLiPPing the switch on Akt and protein kinase C signaling. *Trends Endocrinol Metab* 19, 223-230 (2008).

69. Gao, T., Furnari, F. & Newton, A.C. PHLPP: a phosphatase that directly dephosphorylates Akt, promotes apoptosis, and suppresses tumor growth. *Mol Cell* 18, 13-24 (2005).

70. Pang, X. *et al.* Knockdown of Rhotekin 2 expression suppresses proliferation and induces apoptosis in colon cancer cells. *Oncol Lett* 14, 8028-8034 (2017).

71. Liao, Y.X. *et al.* Silencing of RTKN2 by siRNA suppresses proliferation, and induces G1 arrest and apoptosis in human bladder cancer cells. *Mol Med Rep* 13, 4872-4878 (2016).

72. Wei, W., Chen, H. & Liu, S. Knockdown of Rhotekin 2 expression suppresses proliferation and invasion and induces apoptosis in hepatocellular carcinoma cells. *Mol Med Rep* 13, 4865-4871 (2016).

73. Deryugina, E.I. *et al.* LTBP3 promotes early metastatic events during cancer cell dissemination. *Oncogene* (2018).

74. Hou, Z. *et al.* HBx-related long non-coding RNA MALAT1 promotes cell metastasis via up-regulating LTBP3 in hepatocellular carcinoma. *Am J Cancer Res* 7, 845-856 (2017).
