# Supplementary material for: HOXA13 in etiology and oncogenic potential of Barrett’s esophagus
Source: Nat Commun. 2021 Jun 7;12:3354. doi: 10.1038/s41467-021-23641-8 (PMC8184780; doi:10.1038/s41467-021-23641-8)
Supplement: Supplementary file 7 — Reporting Summary [file 41467_2021_23641_MOESM7_ESM.pdf]

nature research

Corresponding author(s): Maikel P. Peppelenbosch

Last updated by author(s): Feb 18, 2021

Reporting Summary

Nature Research wishes to improve the reproducibility of the work that we publish. This form provides structure for consistency and transparency in reporting. For further information on Nature Research policies, see our Editorial Policies and the Editorial Policy Checklist.

Statistics

For all statistical analyses, confirm that the following items are present in the figure legend, table legend, main text, or Methods section.

n/a	Confirmed	
☐	☒	The exact sample size (*n*) for each experimental group/condition, given as a discrete number and unit of measurement
☐	☒	A statement on whether measurements were taken from distinct samples or whether the same sample was measured repeatedly
☐	☒	The statistical test(s) used AND whether they are one- or two-sided<br>*Only common tests should be described solely by name; describe more complex techniques in the Methods section.*
☐	☒	A description of all covariates tested
☐	☒	A description of any assumptions or corrections, such as tests of normality and adjustment for multiple comparisons
☐	☒	A full description of the statistical parameters including central tendency (e.g. means) or other basic estimates (e.g. regression coefficient) AND variation (e.g. standard deviation) or associated estimates of uncertainty (e.g. confidence intervals)
☐	☒	For null hypothesis testing, the test statistic (e.g. *F*, *t*, *r*) with confidence intervals, effect sizes, degrees of freedom and *P* value noted<br>*Give P values as exact values whenever suitable.*
☐	☒	For Bayesian analysis, information on the choice of priors and Markov chain Monte Carlo settings
☐	☒	For hierarchical and complex designs, identification of the appropriate level for tests and full reporting of outcomes
☐	☒	Estimates of effect sizes (e.g. Cohen's *d*, Pearson's *r*), indicating how they were calculated

Our web collection on statistics for biologists contains articles on many of the points above.

Software and code

Policy information about availability of computer code

Data collection	GEO2R tool (www.ncbi.nlm.nih.gov/geo/geo2r/), R 3.2.3., Biobase 2.30.0, GEOquery 2.40.0, limma 3.26.8
Data analysis	Microsoft Excel 2016, Graphpad Prism 5 and 8, BD FACSDiva v8.0.1, Tophat (version 2.1.0), HTSeq (version 0.6.1p1), R. version 3.2.5 in combination with the module DeSeq2, R version 3.6.3, FIJI version 1.51 (J. Schindelin, et al.<br>Fiji (a version of ImageJ and available since 2008): an open-source platform for biological-image analysis. Nat. Methods, 9 (7) (2012), pp. 676-682), Photoshop CC 2018 version 19.1.9 20190724.r.451 2019/07/24: 1207350 x64.

For manuscripts utilizing custom algorithms or software that are central to the research but not yet described in published literature, software must be made available to editors and reviewers. We strongly encourage code deposition in a community repository (e.g. GitHub). See the Nature Research guidelines for submitting code & software for further information.

Data

Policy information about availability of data

All manuscripts must include a data availability statement. This statement should provide the following information, where applicable:

- Accession codes, unique identifiers, or web links for publicly available datasets
- A list of figures that have associated raw data
- A description of any restrictions on data availability

RNA sequence data is available in Mendeley Data with identifier DOI:10.17632/xxdjv2js5r.1. (https://data.mendeley.com/datasets/xxdjv2js5r/1) 82. There are no restrictions regarding data availability. Supplemental figures, tables, and a macro are included. Source data are provided with this paper and all relevant data are available from the authors.
Datasets used in the manuscript: RNA seq data GSE5758415, GSE6501318, single cell RNA data seq from ref #21 (Owen, R.P. et al) , GSE134520, and GSE81861.

Field-specific reporting

Please select the one below that is the best fit for your research. If you are not sure, read the appropriate sections before making your selection.

- [x] Life sciences
- [ ] Behavioural & social sciences
- [ ] Ecological, evolutionary & environmental sciences

For a reference copy of the document with all sections, see nature.com/documents/nr-reporting-summary-flat.pdf

Life sciences study design

All studies must disclose on these points even when the disclosure is negative.

Sample size	no sample-size calculation was performed; sample sizes were chosen mostly based on the availability of the material.
Data exclusions	Regarding RNA sequencing experiments, genes for which one of the two cell models used had less than ten reads in the control or experimental samples were excluded. The rationale is that a very low expression likely means that gene product has no significant function in that model system. Additionally, such low numbers of reads make any estimate of fold change unreliable. This exclusion criterion was pre-established.
Replication	All cell culture experiments were performed at least three times, fluorescence imaging data was confirmed with anti-GFP IHC staining. All qPCRs were performed in technical duplicates. Multiple techniques and methods were used to verify findings, eg. HOXA13-GFP mouse model and RNA-ISH. Regarding the down-regulation of the chromosome 1 epidermal differentiation complex by HOXA13, two cell lines and both induced expression and knock-out were used. All attempts at replication were successful.
Randomization	Randomization was not performed. Regarding rat trachea in vivo tissue reconstitution model, th use of animals in this study was simply to provide a host for the growth of the xenotransplants. All animals were of the same strain, age and sex. Rat tracheas containing either control or HOXA13 knockout BAR-T cells were implanted into mice that were selected at random from their housing. Mice were then ear tagged to identify whether they were host to a control or HOXA13 knockout xenotransplant. At each of the timepoints, mice containing control or HOXA13 knockout were randomly selected from the appropriate cohort and euthanised to extract the transplant for analysis. No specific technique was used when randomly selecting animals for insertion of the transplant or euthanizing for analysis. Regarding C57BL/6J-Hoxa13-GFP heterozygous mutant mouse model the animals were randomised to the extent that they were not chosen but rather it was what was available as the colony was not big. C57BL/6J wild-type mice (expression along GI tract). Randomization was not performed as there were no treatment groups to which mice could be assigned.
Blinding	Blinding was not performed for the same reasons as described for the randomisation

Reporting for specific materials, systems and methods

We require information from authors about some types of materials, experimental systems and methods used in many studies. Here, indicate whether each material, system or method listed is relevant to your study. If you are not sure if a list item applies to your research, read the appropriate section before selecting a response.

Materials & experimental systems

n/a	Involved in the study	
[ ]	[x]	Antibodies
[ ]	[x]	Eukaryotic cell lines
[x]	[ ]	Palaeontology and archaeology
[ ]	[x]	Animals and other organisms
[ ]	[x]	Human research participants
[x]	[ ]	Clinical data
[x]	[ ]	Dual use research of concern

Methods

n/a	Involved in the study	
[x]	[ ]	ChIP-seq
[ ]	[x]	Flow cytometry
[x]	[ ]	MRI-based neuroimaging

Antibodies

Antibodies used

Antibody; Concentration; Manufacturer; Product #; RRID
1. PE rat anti-mouse (clone 2B11) CD184 (CXCR4); 1:250; BD Pharmingen; 551966; AB_394305;
2. Alexa Fluor 488 rat anti-mouse (clone DECMA-1) anti-CD324 (E-Cadherin); 1:250; Thermo-Fisher Scientific; 53-3249-80; 3. AB_10671270;
4. Anti-human mitochondria (clone 113-1); 1:500; Merck Millipore, Billerica, USA; MAB1273; AB_94052;
5. Mouse anti-human monoclonal (clone OV-TL 12/30) CK7; 1:100; Dako Cytomation, Glostrup, Denmark; M7018; AB_2134589;
6. Mouse anti-human monoclonal (clone 415909) TFF3; 1:50; R&D Systems, Minneapolis, USA; MAB4407; AB_2271768;
7. Rabbit anti-human monoclonal (clone EPR2764Y) CDX2; 1:100; Cell Marque, Rocklin, CA; 235R-14; AB_1516797;
8. Mouse anti-human monoclonal (clone DAK-p63) P63; 1:100; Dako Cytomation, Glostrup, Denmark; M7317; NA;
9. Rabbit anti-human monocolonal (clone EP1601Y) CK5; 1:100; Cell Marque, Rocklin, CA; 305R-16; AB_1159468;

10. Rabbit anti-human Involucrin; 1:100; gift from A/Prof. Pritinder Kaur, Curtin University, Australia; NA; NA;
11. Mouse anti-human Involucrin; 1:500; Sigma-Aldrich, Sigma-Aldrich, St. Louis, Missouri, USA; #I9018; AB_477129;
12.Rabbit polyclonal anti-GFP; 1:100; Merck Millipore, Billerica, USA; #AB3080; AB_91337;
13. Rabbit anti-human KRT5 (clone SP27); 0.51 µg/ml; Ventana, USA; 760-4935; NA;
14. Rabbit anti-human KR7 (clone SP52; 0.536 µg/ml; Ventana, USA; 790-4462; NA;
15. Mouse anti-human P63 (cone 4AU); 0.140 µg/ml; Ventana, USA; 790-4509; NA;
16. Ultramap anti-mouse HRP; NA; Ventana, USA; 760-4313; NA;
17. Ultramap anti-rabbit HRP Ventana; NA; Ventana, USA; 760-4315 NA;

Validation

1. AB_394305; Manufacturer: Flow cytometry (Routinely Tested); The Purified Rat Anti-Mouse CD184 antibody can be used for the immunofluorscent staining and flow cytometric analysis of mouse leukocytes and cell lines that express CXCR4 https://www.bdbiosciences.com/us/applications/research/t-cell-immunology/regulatory-t-cells/surface-markers/mouse/purified-rat-anti-mouse-cd184-2b11cxcr4/p/551852.
Reference PMID:31248780, PMID:31775041, PMID: 8752280

2. AB_10671270; Manufacturer: Applications: IHC, IF, ICC, Flow cytometry, Published species: Artificial Control, Human, Mouse. Applications Tested: This DECMA-1 antibody has been tested by immunocytochemistry on formaldehyde fixed and permeabilized MDCK cells and can be used at less than or equal to 20 µg/mL. Reference: PMID:11329369, PMID:2419126, PMID: 25241035
https://www.thermofisher.com/order/genome-database/dataSheetPdf?producttype=antibody&productsubtype=antibody_primary&productId=53-3249-80

3. AB_94052; Manufacturer: application: IF, IHC, IP; Gives mitochondrial staining on all human cell types and does not cross react with rat and mouse tissue. The staining pattern is best described as a spaghetti-like staining pattern in fibroblasts and a perinuclear large speckled pattern in lymphonoid cells. Quality: routinely evaluated by immunohistochemistry on heart ventricle cells reference: (more than 190) PMID:29732581, PMID:30759398, PMID:30840878 https://www.sigmaaldrich.com/catalog/product/mm/mab1273?lang=en&region=NL

4. AB_2134589; Manufacturer: Optimized for immunohistochemistry (IHC) with validated protocols. Reference: PMID:24189144, PMID:24605829, PMID:28541534, PMID:30551188, PMID:30715257, PMID:31018139, PMID:31290979, PMID:32080251
https://www.agilent.com/en/product/immunohistochemistry/antibodies-controls/primary-antibodies/cytokeratin-7-(concentrate)-76620#productdetails

5. AB_2271768; Manufacturer: Immunocytochemistry; Immunohistochemistry; Western Blot. Does not cross react with recombinant human TFF2. Reference: PMID: 19651633, PMID: 18682706
https://www.rndsystems.com/products/human-trefoil-factor-3-antibody-415909_mab4407#product-datasheets

6. AB_1516797; Manufacturer: The stated primary antibody may be used as the primary antibody for immunohistochemical staining of formalin-fixed, paraffin-embedded tissue sections. This antibody is intended for in vitro diagnostic (IVD) use (IHC). Colon adenocarcinoma (control) is highlighted by CDX-2 antibody in a strong nuclear staining reaction. Tested on multiply tissues.
https://www.cellmarque.com/antibodies/CM/2025/CDX-2_EPR2764Y
file://storage.erasmusmc.nl/m/MyDocs/039119/My%20Documents/Desktop/NC_revision%203/New_version_of_manuscript_and_supportive_files/CDX-2%20(EPR2764Y)_CM_MAN_EN_IVD_6.0.pdf

7. AB_2755007; Manufacturer: for immunohistochemistry.Reference: PMID:31319607
https://www.agilent.com/store/en_US/Prod-M731701-2/M731701-2?navAction=push&catId=SubCat3ECS_86543&pCatName=Primary%20Antibodies

8. AB_1159468; Manufacturer: For In Vitro Diagnostic Use (IVD) (IHC). Tested on multiply tissues.
https://www.cellmarque.com/antibodies/CM/1990/Cytokeratin-5_EP1601Y

10. AB_477129; Manufacturer: Monoclonal Anti-Involucrin may be used for the localization of involucrin using various immunochemical assays such as IHC, IP, ELISA, WB. The antibody does not react with mouse epidermis, permitting use in studies of human xenografts in nude mice. The antibody stains the upper spinous and granular layers in human skin and the cytoplasm of suprabasal terminally differentiated keratinocytes in stratified colonies.
Reference: PMID:28332183, PMID: 15840658, PMID: 9573271
https://www.sigmaaldrich.com/catalog/product/sigma/i9018?lang=en&region=NL
https://www.sigmaaldrich.com/content/dam/sigma-aldrich/docs/Sigma/Datasheet/2/i9018dat.pdf

11. AB_91337; Manufacturer: This Anti-Green Fluorescent Protein Antibody is validated for use in ELISA, IC, IH, WB for the detection of Green Fluorescent Protein.
Reference: PMID:17348003, PMID:18459137, PMID:21452242, PMID:23124714, PMID:23224860, PMID:25043553, PMID:25976033, PMID:26357589, PMID:26937712, PMID:27159642, PMID:27637097, PMID:27707971, PMID:28472856, PMID:28703706, PMID:28803542, PMID:29175956, PMID:30255935, PMID:30295607, PMID:30297418, PMID:30308166, PMID:31165473, PMID:31396959, PMID:32609582, PMID:32639229
https://www.merckmillipore.com/NL/en/product/Anti-Green-Fluorescent-Protein-Antibody,MM_NF-AB3080?ReferrerURL=https%3A%2F%2Fwww.google.com%2F&bd=1
12. Cytokeratin 5 (SP27) Rabbit Monoclonal Primary Antibody. Cytokeratin 5 (SP27) antibody is intended for qualified laboratories to qualitatively identify by light microscopy the presence of associated antigens in sections of formalin-fixed, paraffin-embedded tissue sections using IHC test methods. Use of this antibody is indicated, subsequent to clinical differential diagnosis.

http://ww.ventanadiscovery.com/product/1797?type=2288

13. CONFIRM anti-Cytokeratin 7 (SP52) Rabbit Monoclonal Primary Antibody. Manufacturer: This antibody may be used to aid in the identification of normal and neoplastic cells of ovary, lung and breast epithelial origin which express Cytokeratin 7, and those of colonic and prostate epithelial lineage which lack Cytokeratin 7 production. The antibody is intended for qualitative staining in sections of formalin fixed, paraffin embedded tissue. This product should be interpreted by a qualified pathologist in conjunction with histological examination, relevant clinical information and proper controls. This antibody is intended for in vitro diagnostic (IVD) use. http://ww.ventanadiscovery.com/product/71?type=66

14. VENTANA anti-p63 (4A4) Mouse Monoclonal Primary Antibody. Manufacturer: The antibody is intended for qualitative staining in sections of formalin fixed, paraffin embedded tissue. This product should be interpreted by a qualified pathologist in conjunction with histological examination, relevant clinical information and proper controls. This antibody is intended for in vitro diagnostic (IVD) use. http://ww.ventanadiscovery.com/product/1529?type=1986
Regarding Rat trachea in vivo tissue reconstitution model. The antibodies were validate by using positive and negative tissue controls i.e. tissues known to express or not express the antigen, respectively (CK5, TFF3, CDX2, p63, CK7, involucrin, anti-human mitochondria). Antibodies used for multiplex staining were titrated and validated with positive tissue (p63, CK5, CK7, Ultramap anti-mouse HRP, Ultramap anti-rabbit HRP Ventana). 9,15,16 NA

Eukaryotic cell lines

Policy information about cell lines

Cell line source(s)	1. EPC2-hTERT gift of K.K. Krishnadath, University of Amsterdam, The Netherlands<br>2.Het-1A was a gift of J.W.P.M. van Baal, University Utrecht, The Netherlands<br>3. BAR-T was a gift of dr. J.W.P.M. van Baal who had, in turn, received them from dr. R.F. Souza,University of Texas Southwestern Medical Center, USA<br>4. KH2 mESCs were a gift of J. Gribnau, Erasmus MC University Medical Center Rotterdam, The Netherlands<br>5. Swiss albino cells was a gift of J.W.P.M. van Baal, University Medical Center Utrecht, The Netherlands<br>6. HEK293T were originally from ATCC<br>7. M15 gift of N. Hastie, University of Edinburgh, UK
Authentication	Identity of HET1A and EPC2-hTERT cell lines was confirmed with short tandem repeats (STR) analysis by DSMZ. for other cell lines, authentication was not performed.
Mycoplasma contamination	All cells were regularly checked for mycoplasma by handing in samples prepared according to instructions at GATC Biotech (Konstanz, Germany). All cell lines tested negative for mycoplasma contamination.
Commonly misidentified lines (See ICLAC register)	checked, none

Animals and other organisms

Policy information about studies involving animals; ARRIVE guidelines recommended for reporting animal research

Laboratory animals	Mus musculus. For the RNA expression analysis throughout the murine gastrointestinal tract, four C57BL/6J wildtype mice were used, two males of three months old and two females of five months old. For the heterozygous mutant mouse model C57BL/6J-Hoxa13-GFP mice were used, two females of five months old. Mice were generally kept with 12:12 hours light - dark, the animal room temperature is between 20 and 24oC and the relative humidity is 55±10%. NOD SCID gamma mice were housed in microisolator cages with a 14hr light/10hr dark cycle, standard chow and water ad libitum, and temperature and humidity maintained at 21±1°C and 50±10%, respectively.
Wild animals	no wild animals were used in the study
Field-collected samples	no field collected samples were used in the study
Ethics oversight	Murine experiments were approved by the Ethical Committee for Animal Experiments of the Erasmus MC and were performed according to the guidelines of the same institution. In addition, murine experiments were approved by the Peter MacCallum Cancer Centre Animal Experimentation Ethics Committee and were performed according to the guidelines of the same institution.

Note that full information on the approval of the study protocol must also be provided in the manuscript.

Human research participants

Policy information about studies involving human research participants

Population characteristics	Regarding levels of HOX genes along the GI tract, included patients had unexplained symptoms, mostly anemia, while inflammatory bowel disease patients were excluded. Regarding Barrett's biopsies, patients had to have confirmed Barrett's esophagus. Regarding the biopsies from the cecum, patients had to be without diagnosis or aberrant findings during colonoscopy. All identifiable data were removed from the samples used for the optimal protection of patient privacy and as we had not planned any analysis including these parameters.

Recruitment	Patients visiting the endoscopy unit of the Erasmus University Medical Center, department of Gastroenterology & Hepatology were asked to participate, no financial or other compensation was offered, participation was voluntary. any potential self-selection bias or other biases that may be present and how these are likely to impact results are known to us.
Ethics oversight	The use of these samples was approved by the Erasmus MC medical ethical committee (MEC-2015-208, MEC-2015-209, MEC-2015-199, MEC-2010-093; tissues were handled according to the FEDERA code of conduct and informed consent was obtained.

Note that full information on the approval of the study protocol must also be provided in the manuscript.

Flow Cytometry

Plots

Confirm that:

- [x] The axis labels state the marker and fluorochrome used (e.g. CD4-FITC).
- [x] The axis scales are clearly visible. Include numbers along axes only for bottom left plot of group (a 'group' is an analysis of identical markers).
- [x] All plots are contour plots with outliers or pseudocolor plots.
- [x] A numerical value for number of cells or percentage (with statistics) is provided.

Methodology

Sample preparation	Cells were double stained with 0.8 µg PE Rat Anti-Mouse CD184 (CXCR4) and 2.0 µg Anti-CD324 Alexa Fluor® 488 (E-Cadherin) at 4 °C for 45 min, washed and subsequently sorted.
Instrument	BD FACSCantoTM II (BD Biosciences, USA).
Software	Data were analyzed with BD FACSDiva v8.0.1 software, which was obtained from BD Biosciences, and processed using Microsoft Excel.
Cell population abundance	The result of the sort was investigated by quantification of other definitive endoderm markers, such as Sox17 and Foxa1, in addition to CXCR4 and E-cadherin. Pluripotency markers such as Nanog were also quantified (see Extended data table 1). Additionally, ingenuity pathway analysis (IPA), was used to investigate association with the list of genes known to be involved in the differentiation of embryonic cells. Population of CXCR4+ E-Cad+ cells was 27 +/-16%.
Gating strategy	FSC/SSC gates were used to exclude cellular or other debris and gate for single cells. CXCR4+/E-cadherin+ positivity was determined at 700 and 500 fluorescence intensity units based on the signal strength of control cells. negative for both markers. Double positive cells were counted and sorted.

- [x] Tick this box to confirm that a figure exemplifying the gating strategy is provided in the Supplementary Information.
